# Identification of novel CD44v6-binding peptides that block CD44v6 and deliver a pro-apoptotic peptide to tumors to inhibit tumor growth and metastasis in mice

**DOI:** 10.7150/thno.50564

**Published:** 2021-01-01

**Authors:** Fatima Khan, Smriti Gurung, Gowri Rangaswamy Gunassekaran, Sri Murugan Poongkavithai Vadevoo, Lianhua Chi, Uttapol Permpoon, Md. Enamul Haque, Yun-Ki Lee, Soo-Woong Lee, Soyoun Kim, Byungheon Lee

**Affiliations:** 1 Department of Biochemistry and Cell Biology, School Medicine, Kyungpook National University, 680 Gukchaebosang-ro, Jung-gu, Daegu 41944, Republic of Korea.; 2 BK21 Plus KNU Biomedical Convergence Program, Department of Biomedical Science, School Medicine, Kyungpook National University, 680 Gukchaebosang-ro, Jung-gu, Daegu 41944, Republic of Korea.; 3 CMRI, School Medicine, Kyungpook National University, 680 Gukchaebosang-ro, Jung-gu, Daegu 41944, Republic of Korea.

**Keywords:** CD44v6, c-Met, metastasis, peptide, phage display

## Abstract

CD44v6, a splice variant of the cell surface glycoprotein CD44, acts as a co-receptor for c-Met and is upregulated in tumors with high metastatic potential.

**Methods:** We screened a phage-displayed peptide library for peptides that selectively bind to CD44v6-overexpressing cells and exploited them to block CD44v6 and deliver a pro-apoptotic peptide to tumors for cancer therapy.

**Results:** CNLNTIDTC (NLN) and CNEWQLKSC (NEW) peptides bound preferentially to CD44v6-high cells than to CD44v6-low cells. The binding affinities of NLN and NEW to CD44v6 protein were 253 ± 79 and 85 ± 18 nM, respectively. Peptide binding to CD44v6-high cells was inhibited by the knockdown of CD44v6 gene expression and competition with an anti-CD44v6 antibody. A pull-down assay with biotin-labeled peptides enriched CD44v6 from cell lysates. NLN and NEW induced CD44v6 internalization and inhibited hepatocyte growth factor-induced c-Met internalization, c-Met and Erk phosphorylation, and cell migration and invasion. In mice harboring tumors, intravenously administered NLN and NEW homed to the tumors and inhibited metastasis to the lungs. When combined with crizotinib, a c-Met inhibitor, treatment with each peptide inhibited metastatic growth more efficiently than each peptide or crizotinib alone. In addition, KLAKLAKKLAKLAK pro-apoptotic peptide guided by NLN (NLN-KLA) or NEW (NEW-KLA) killed tumor cells and inhibited tumor growth and metastasis. No significant systemic side effects were observed after treatments.

**Conclusions:** These results suggest that NLN and NEW are promising metastasis-inhibiting peptide therapeutics and targeting moieties for CD44v6-expressing metastases.

## Introduction

CD44, a cell surface glycoprotein, mediates contact between the cell and the extracellular matrix and regulates cell differentiation, migration, and behavior; consequently, this glycoprotein is essential for maintaining tissue integrity 1, 2. Certain isoforms of CD44, such as CD44 variant 6 (CD44v6), are expressed on cancer cells with high metastatic potential and stem cell-like characteristics 2, 3. Elevated CD44v6 expression is largely restricted to advanced-stage malignancies and is more prevalent in metastatic than in non-metastatic cancers 4, 5. Patients with CD44v6-positive tumors have a poorer prognosis than those with CD44v6-negative tumors 6, 7. CD44v6 is required for the interaction between c-Met receptor tyrosine kinase and hepatocyte growth factor (HGF), which plays critical roles in tumor metastasis 8, 9. HGF can activate c-Met in a CD44v6-dependent manner 8, 10, whereas CD44v6 can activates c-Met independently of HGF 11, 12. The concomitant expression of CD44v6 and c-Met and their cooperative activities in cancer cells empowers these cells to metastasize and thus severely affect the patient's prognosis 9, 13. CD44v6- and c-Met-expressing tumor cells are protected against drug-induced apoptosis 14. Moreover, the activation of vascular endothelial growth factor receptor-2 (VEGFR-2), an angiogenic factor, is also dependent on CD44v6 15.

Researchers and clinicians have exploited antibodies and peptides to block the interaction between CD44v6 and c-Met and inhibit tumor growth 6, 7. Anti-CD44v6 antibodies such as bivatuzumab block signaling downstream of c-Met and have been used as cancer treatments 15, 16. The combination of bivatuzumab with mertansine, a microtubule inhibitor, has been used to treat patients with head and neck cancer 17, 18. However, clinical trials of this combination were discontinued because of severe skin toxicities, including one fatal outcome, that were attributed to the mertansine conjugates. Unlike antibodies, peptides are generally subject to rapid (i.e., within several hours) renal excretion and are also vulnerable to degradation by peptidases 19, 20. Nevertheless, peptides have competitive advantages over antibodies, including a smaller size, superior deep tissue penetration, more rapid accumulation at target tissues, and easier quality control during chemical synthesis, lower production costs, and a reduced risk of immunogenicity 21-23. A multi-species mutational analysis of the exon v6 region of CD44v6 identified the five-amino-acid peptides NRWHE (human), NGWQG (mouse), and NEWQG (rat) as essential determinants of the interaction between CD44v6 and c-Met 24. The CD44v6-derived peptides inhibited c-Met-dependent cell migration 24 and vascular endothelial growth factor-induced activation of VEGFR-2 15. In addition to mutational or structural approaches, researchers have used phage-displayed random peptide library screening to identify peptides that selectively bind to CD44v6. The PFTVSVPFVWNFTAD peptide was identified by screening a phage library against a v6 domain of CD44v6 protein, and this peptide was shown to bind to CD44v6-high prostate tumor cells 25. The THENWPA peptide was selected by screening random peptides against purified CD44v3-v10 protein and was shown to bind to gastric tumor cells 26.

In this study, we aimed to identify novel CD44v6-binding peptides in different ways: first, we screened phage-displayed random peptide library to select peptides that bind to and subsequently block CD44v6; second, we used CD44v6-expressing cells for the screening as we hypothesized that CD44v6 expressed on a live cell membrane would have a more natural conformation than recombinant, purified protein; third, we exploited the CD44v6-binding peptides as a targeting moiety to guide a pro-apoptotic peptide to tumors and subsequently inhibit tumor growth and metastasis.

## Materials and Methods

### Cell cultures

HEK 293T human embryonic kidney cells, MDA-MB231 human breast tumor cells, MCF-7 human breast tumor cells, 4T1 mouse breast tumor cells, and Panc-1 human pancreatic tumor cells were purchased from American Type Culture Collection (ATCC, Rockville, MD). MDA-MB231-luc and 4T1-luc that express luciferase were obtained from Perkin Elmer (Waltham, MA). All cells were grown in Dulbecco's modified Eagle's medium (DMEM, HyClone, South Logan, UT) except MCF-7 cells, which were grown in Roswell park memorial institute medium (RPMI, HyClone). All media were supplemented with 10% fetal bovine serum (FBS, ThermoFisher Scientific, Waltham, MA). Cells were maintained at 37 °C in a humidified atmosphere containing 5% CO_2_.

### Transient transfection and preparation of CD44v6-overexpressing cells

The green fluorescent protein (GFP)-tagged mammalian expression vector pCMV6-AC-GFP containing CD44v6 open reading frame was purchased from Origene (catalogue no. RG234071, Rockville, MD). HEK 293T cells were seeded into 6-well plates at a density of 2 × 10^5^ cells/well and incubated for 24 h to reach 60%-70% confluence. Ten microliters of Lipofectamine 2000 reagent (ThermoFisher Scientific) were diluted in 250 µL with Opti-MEM (ThermoFisher Scientific) and incubated with 4 µg of the expression vector for 30 min at room temperature. Subsequently, cells were incubated with this DNA-transfection reagent mixture for 4-6 h. The mixture was replaced with culture medium containing 10% FBS, and the cells were incubated for an additional 48 h.

### Screening of a phage peptide library using CD44v6-expressing cells

The T7 415-1b phage vector was purchased from Novagen (Madison, WI) and used to construct a phage library displaying CX7C (C, cysteine; X7, seven random amino acids in which hydrophobic amino acids were enriched to occur at a frequency of at least one per every seven residues). The library had a diversity of approximately 1 × 10^9^ plaque-forming units (pfu)/mL. Phages (1 × 10^9^ pfu) were then incubated with CD44v6 expression vector-transfected HEK 293 cells at 4 °C for 1 h. Phages bound to the cells were eluted by incubation with 500 µL of a suspension of BL21 host bacteria for 10 min at room temperature. To remove phages that had bound non-specifically to cells, the eluted phages were then incubated with non-transfected parental HEK 293T cells at 4 °C for 30 min. Unbound phages in the supernatant were collected, diluted in 10 mL of LB broth, and amplified by culture with BL21 host bacteria. The amplified phages were used in the next round of the screening cycle. A total of 60 phage clones were selected after the third, fourth, and fifth rounds of screening with the transfected cells, and 10 phage clones were selected after the fifth round of screening with non-transfected cells. DNA inserts in the selected phage clones were subjected to a sequencing analysis by Macrogen Inc. (Seoul, Korea). The corresponding amino acid sequences were aligned and analyzed using the Clustal W program to identify shared amino acid sequences or consensus motifs.

### Synthesis and analysis of peptides

CNLNTIDTC (M.W. = 995.4), CNEWQLKSC (M.W. = 1110.5), CNLNTIDTCGGGKLAKLAKKLAKLAK (M.W. = 2672.7), and CNEWQLKSCGGGKLAKLAKKLAKLAK (M.W. = 2786.8) peptides were synthesized and purified via high-performance liquid chromatography (HPLC) to >90% purity, and the masses were confirmed via MALDI-TOF performed by Anygen Inc. (Gwangju, Korea) (Figure S1A-D, respectively). CNLNTIDTC and CNEWQLKSC were conjugated with fluorescein isothiocyanate (FITC), 5-carboxytetramethylrhodamine (TAMRA), Flamma 675 near-infrared (NIR) fluorescence dye (BioActs, Incheon, Korea), or biotin at the amino-termini. The NSSSVDK peptide, a peptide sequence present in the phage coat protein, was used as a control peptide.

### Phage cell-binding ELISA

Cells were plated in 96-well plates at a density of 5 × 10^3^ cells per well and blocked with 5 mg/mL bovine serum albumin (BSA) at room temperature for 1 h. After washing, 100 μL suspension of each phage clone (1 × 10^9^ pfu) were added to the well and incubated at 4 °C for 1 h. After washing, the cells were incubated with a horseradish peroxidase-conjugated anti-T7 tail fiber antibody (Merck, Kenilworth, NJ) diluted in the blocking buffer (1:10,000) at room temperature for 1 h. After washing, cells were incubated with a 3,3ʹ,5,5ʹ-tetramethylbenzidine substrate (Pierce, Rockland, IL) for 10-15 min. The reaction was stopped by adding 100 μL of 2 M sulfuric acid, and the absorbance was read at 450 nM using a microplate reader (Tecan, Zurich, Switzerland).

### Fluorescence microscopic analyses of peptide binding

Cells were seeded in each well of an 8-well chamber slide at a density of 1 × 10^5^ cells per well, incubated overnight, and then treated with 1% BSA at room temperature for 1 h. Subsequently, the cells were incubated with FITC-conjugated peptides (25 µM) at 4 °C for 1 h, blocked with BSA, fixed, and incubated with an anti-human CD44v6 mouse monoclonal antibody (clone no. VFF-7, 1:100 dilution; Santa Cruz Biotechnology, Dallas, TX) or a rabbit anti-CD44v6 polyclonal antibody (1:100 dilution; Merck). The cells were then incubated with an Alexa Fluor 594-labeled goat anti-mouse IgG or goat anti-rabbit IgG secondary antibodies (Merck). After incubation, the cells were counterstained with 4ʹ,6-diamidino-2-phenylindole (DAPI, Sigma-Aldrich, St. Louis, MI), mounted with ProLong antifade reagent (Thermo Fischer Scientific), and observed under a confocal microscope (Zeiss, Jena, Germany).

### Flow cytometric analyses of peptide binding and competition assays

Cells (1 × 10^5^ cells) were harvested and suspended in culture medium containing 1% BSA for 1 h, followed by an incubation with FITC-labeled peptides (25 µM) at 4 °C for 1 h. After washing, the cells were analyzed using a FACSCalibur flow cytometer (BD, Franklin Lakes, NJ). For competition assays, cells were incubated with 5 µg/mL of an anti-human CD44v6 mouse monoclonal antibody (clone no. VFF-18, 1:100 dilution; Abcam, Cambridge, MA) and anti-human CD44 monoclonal antibody (clone no. 156-3C11, 1:100 dilution; ThermoFisher Scientific) for 1 h to compete with peptide binding, followed by an incubation with FITC-labeled peptides (10 µM) before analysis on a flow cytometer (BD). In another set of assays, cells were incubated with peptides (50 µM) for 1 h to compete with antibody binding, followed by an incubation with 5 µg/mL of the anti-human CD44v6 mouse monoclonal antibody for 1 h, The cells were then incubated with an Alexa Fluor 488-labeled goat anti-mouse IgG secondary antibody (Merck) before analysis on a flow cytometer (BD).

### Surface plasmon resonance (SPR) analysis of protein binding affinity

The binding affinity (K_D_) of CD44v6-binding peptides to the CD44v6 protein was measured using an SPR instrument (Reichert Technologies, Depew, NY). A streptavidin chip was used as the immobilization substrate and exposed to biotin-labeled peptides (25 μΜ) at 5 μL/min for 50 min. The streptavidin only surface was used for the reference surface. All SPR signals were corrected by subtraction with the response of the reference surface. After washing, the chip was flowed with a solution of CD44v6-Fc or CD44-Fc protein (R&D Systems, Minneapolis, MN) in binding buffer (137 mM NaCl, 10 mM phosphate, 2.7 mM KCl pH 7.4, 62.5 µM BSA, and 0.005% Tween-20) at concentrations of 0.125, 0.25, 0.5, 1, and 2 μM at 10 μL/min for 3 min. Five concentrations (0.125, 0.25, 0.5, 1, and 2 µM) of CD44v6-Fc and CD44-Fc were used in a single-cycle dynamic SPR measurements. Resonance units were recorded and analyzed using Scrubber 2.0 software (BioLogic Software, Campbell, Australia). K_D_ values were calculated by analyzing the steady-state binding data by fitting the curve of binding level against concentration with a 1:1 binding model (GraphPad Prism 7.0 software).

### Knockdown assays

CD44v6 gene expression was knocked down using CD44v6 small interfering RNAs (siRNAs) as previously described 27. Two CD44v6-specific siRNAs (v6-1: 5ʹ-AGU ACA ACG GAA ATT-3ʹ and v6-2: 5ʹ-GGA UAU CGC CAAACA CCC ATT-3ʹ) were synthesized by Bioneer (Daejeon, Korea). Control siRNA (5ʹ-CUA CGC CAA UUU CGU (dTdT)-3ʹ) were purchased from Bioneer. MDA-MB231 cells were transfected twice with a mixture of the CD44v6 siRNAs in Lipofectamine 2000 at an interval of 24 h. At 24-72 h post-transfection, the cells were lysed and subjected to Western blotting analysis using antibodies against CD44v6 and CD44. After 24 h of CD44v6 knockdown, cells were subjected to the peptide binding assays using a flow cytometer (BD) and confocal microscope (Zeiss) as described above.

### Pull-down assays

Biotin-labeled peptides were incubated with streptavidin magnetic beads (Bioclone Inc., San Diego, CA) at room temperature for 30-60 min under gentle rotation. MDA-MB231 cells were then lysed using a cell lysis reagent (ThermoFisher Scientific) containing protease inhibitors. The lysates were incubated with the biotinylated peptide (25 µM) and streptavidin bead complexes at room temperature for 30-60 min under gentle rotation. In another set of assays, the recombinant human CD44v6 protein was used instead of the cell lysates. The complexes were pulled down and incubated with an elution buffer containing 2 mM D-biotin (Bioclone Inc.) for 5-10 min, and the eluates were collected and subjected to electrophoresis.

### Internalization of CD44v6 and c-Met

To examine the internalization of CD44v6, 1 × 10^5^ MDA-MB231 cells were seeded in each well of an 8-well chamber slide and cultured for 24 h. Next, the cells were incubated with a fresh culture medium containing FITC-labeled peptides (10 μM) at 37 °C for 10, 30, and 60 min and then stained with an anti-human CD44v6 mouse monoclonal antibody (clone no. VFF-7; Santa Cruz Biotechnology). The cells were then incubated with an Alexa Fluor 594-labeled goat anti-mouse IgG secondary antibody (Merck). To examine the internalization of c-Met, cells were incubated with recombinant human HGF at 25 ng/mL (R&D Systems, Minneapolis, MN) for 30 or 60 min with or without pre-treatment with peptides (20 µM) for 10 min. After incubation, the cells were stained with an anti-Met rabbit polyclonal antibody (clone no. C-28, 1:100 dilution; Santa Cruz Biotechnology). The cells were then incubated with an Alexa Fluor 594-labeled goat anti-rabbit IgG secondary antibody (Merck). For nuclear staining, the cells were incubated with DAPI and the ProLong antifade reagent before observation under a confocal microscope (Zeiss).

### Western blotting and phosphorylation analysis

To examine CD44v6 and CD44 protein levels, cell lysates were subjected to Western blotting analyses using an anti-human CD44v6 mouse monoclonal antibody (clone no. VFF-7; Santa Cruz Biotechnology) and anti-CD44 mouse monoclonal antibody (clone no. DF1485, 1:1000 dilution; Santa Cruz Biotechnology). A control, glyceraldehyde 3-phosphate dehydrogenase (GAPDH) was examined using an anti-GAPDH antibody (clone no. G-9; Santa Cruz Biotechnology).

To examine c-Met and Erk phosphorylation, MDA-MB231 cells were serum-starved for 24 h, pre-treated with CD44v6-binding peptides (20 µM) for 10 min, and incubated with 25 ng/mL HGF for 10 min. After the incubation, the cell lysates were subjected to Western blotting analyses using an anti-phospho-Met rabbit monoclonal antibody (clone no. D26; Cell Signaling Technology), anti-Met mouse monoclonal antibody (clone no. 25H2, 1:1000 dilution; Cell Signaling Technology), anti-phospho-Erk1/2 rabbit monoclonal antibody (clone no. 197G2; Cell Signaling Technology), and anti-ERK1 rabbit polyclonal antibody (1:1000 dilution; Santa Cruz Biotechnology). The labeled bands on immunoblots were detected using enhanced chemiluminescence reagents (ThermoFisher Scientific).

### Transwell cell migration and invasion assays

Transwell chambers (24-wells) with 8 μm pores were obtained from Corning (Corning, NY). MDA-MB231 and 4T1 cells (1 × 10^6^ cells) were harvested, resuspended in 200 μL of DMEM containing CD44v6-binding peptides (20 µM), and then seeded into the upper chamber of the transwell chambers. The lower chamber was filled with 600 μL of DMEM containing 25 ng/mL HGF. After 24 h of incubation, cells that migrated into the reverse side of the transwell membrane were fixed with methanol, stained with crystal violet, and then counted under a light microscope. An average of five visual fields was obtained. For invasion assays, the transwell membrane was pre-coated with 300 µg/mL of Matrigel (Corning).

### Cytotoxicity and apoptosis assays

Cells were seeded into 96-well plates at a density of 5 × 10^3^ cells per well and incubated with different concentrations of peptides (0, 2, 4, 8, 16, 32, 64, 128, and 256 µM) at 37 °C for 24 h. After incubation, 10 µl of CCK-8 dye (Dojindo, Kumamoto, Japan) were added to each well in 100 µl of culture medium. The absorbance in each well was measured using a microplate reader (Tecan) at a wavelength of 450 nm.

For apoptosis assays, cells were plated in 6-well plates at a density of 5 × 10^5^ cells per well overnight and treated with different concentrations of peptides (0, 2, 4, 8, 16, 32, 64, 128, and 256 µM) at 37 °C for 2 h. Cells were harvested, washed, and mixed with annexin V binding buffer. Then, 1 μg/mL of propidium iodide and 5 μL of APC annexin V reagent (ThermoFisher Scientific, Waltham, MA) were added to stain necrotic and apoptotic cells. Percent of annexin V-positive and propidium iodide-negative cells were measured using flow cytometry.

### Intracellular trafficking of peptides using MitoTracker and LysoTracker

To examine the intracellular trafficking of peptides cells were incubated with peptides (10 µM) at 37 °C for 2 h and stained with MitoTracker Deep Red FM (ThermoFisher Scientific), a mitochondrial marker. In another set of experiments, cells were incubated with peptides at 37 ºC for 4 h and stained with LysoTracker Red DND-99 (ThermoFisher Scientific), a late endosome/lysosome marker. After staining, cells were fixed with 4% paraformaldehyde for 10 min at room temperature, stained with DAPI, and then mounted using ProLong gold anti-fade reagent (ThermoFisher Scientific). Subcellular fluorescence was detected using a confocal microscope (Zeiss).

### *In vivo* whole-body and *ex vivo* fluorescence imaging of tumor homing of peptides

Mice for animal experiments were purchased from Orient Bio Inc. (Seongnam, Korea) and maintained in conformance with the Guidelines of the Institutional Animal Care and Use Committee of Kyungpook National University (permission no. 2014-1-121). A total of 1 × 10^6^ MDA-MB231 cells were subcutaneously injected into the right flank of each 6-week-old female BALB/c nude mouse. When the tumor reached a volume of approximately 100 mm^3^, the mouse was injected intravenously with Flamma 675 NIR fluorescence dye-labeled peptides (1 mg/kg body weight). Whole-body fluorescence imaging was performed under inhalational anesthesia using an IVIS imaging system (Perkin Elmer). After *in vivo* imaging, each mouse was euthanized, the tumor and control organs (liver, kidney, spleen, heart, and lung) were isolated, and *ex vivo* images were obtained using the IVIS imaging system.

### Anti-tumor therapy using experimental tumor metastasis model

A mouse model of lung metastasis of breast cancer was prepared by injecting 1 × 10^6^ MDA-MB231-luc cells into 6-week-old female BALB/c nude mice via tail vein. To monitor the localization of the tumor cells in the lungs, the mice were injected intraperitoneally with D-luciferin at a dose of 150 mg/kg body weight and subjected to a whole-body bioluminescence imaging using IVIS imaging system (Perkin Elmer) after a 10-min resting period. Mice (*n* = 10 per group) were randomly assigned to groups based on the luminescence intensity. At 1 h after tumor cell injection, tumor-bearing mice received intravenous injections of CD44v6-binding peptides through the tail vein (14.2 mg/kg body weight, thrice weekly for 3 weeks) alone or in combination with orally administered crizotinib in 5% dimethyl sulfoxide (DMSO) (25 mg/kg of body weight, twice weekly for 3 weeks) as previously described 28, 29. Metastatic tumor growth after treatments was monitored by measuring the total photon flux (number of photons/second) in the whole body using the IVIS imaging system. The body weights of mice and tumor ulceration were monitored throughout the treatment period. At the end of the treatment period, half of the mice (*n* = 5 per group) were used for the collection of blood, sacrificed, the lungs were harvested and weighed, and the numbers of metastatic tumor nodules in the lungs were counted. The remaining mice (*n* = 5 per group) were maintained until death to determine the survival rates. For the analysis of hematological parameters, 1 mL of blood was collected from each mouse and separated into 500 µL aliquots used to prepare serum and plasma. Serum was obtained by centrifuging clotted blood at 4 °C twice, followed by filtration (pore size: 0.22 µm). Plasma was obtained by centrifuging ethylenediaminetetraacetic acid-treated samples. Hematological parameters and liver and kidney function markers were measured by DGMIF (Daegu, Korea).

### Anti-tumor therapy using spontaneous tumor metastasis model

4T1-luc cells (1 × 10^6^ cells) were orthotopically inoculated into the mammary fat pads in 6-week-old female BALB/c mice. Panc-1 cells (1 × 10^6^ cells) were subcutaneously inoculated into lower right flank in 6-week-old female BALB/c nude mice To monitor 4T1-luc tumor growth, mice were intraperitoneally injected with D-luciferin (150 mg/kg body weight), incubated for 10 min, and subjected to a whole-body bioluminescence imaging using the IVIS imaging system (Perkin Elmer). At two weeks after the inoculation, mice were randomly sorted into groups based on the luminescence intensity (4T1-luc) or tumor size (Panc-1). CD44v6-binding peptides were administered intravenously to 4T1 tumor-bearing mice (*n* = 6 per group) through the tail vein (14.2 mg/kg body weight, thrice weekly for three weeks) alone or in combination with crizotinib. Crizotinib in 5% DMSO was administered orally (12.5 and 25 mg/kg body weight, twice weekly for three weeks). CD44v6-targeted pro-apoptotic peptides or CD44v6-binding peptides and pro-apoptotic peptide in combination were intravenously administered to 4T1 or Panc-1 tumor-bearing mice (*n* = 10 per group) through the tail vein (10 mg/kg body weight, thrice weekly for 3 weeks). Primary tumor growth was monitored by measuring tumor volumes using a caliper and total photon flux in the whole body using the IVIS imaging system. The body weights and tumor ulceration were monitored throughout the treatment period. At the end of the treatments, half of the mice were used for the collection of blood, sacrificed, and the primary tumor weights and numbers of metastatic tumor nodules in the isolated lungs were determined. The remaining mice were maintained until death to determine the survival rates. The hematological parameters and liver and kidney function markers in plasma and serum samples were analyzed as described above. The normal ranges of hematological parameters of the BALB/c mouse and BALB/c nude mouse were obtained from Charles River Laboratories website (www.criver.com).

### Immunohistochemistry of tumor tissues

To examine the location of peptides in respect for CD44v6 at tumor tissues, tumors were isolated from mice intravenously injected with the fluorescence dye-labeled peptides for tumor homing, subjected to *ex vivo* imaging, and then fixed overnight with 4% paraformaldehyde and rapidly frozen. Tissue sections (8-μm thick) were prepared and then stained with an anti-human CD44v6 mouse monoclonal antibody (clone no. VFF-7, 1:50 dilution; Santa Cruz Biotechnology) and subsequently with an Alexa Fluor 594-labeled goat anti-mouse IgG antibody. The nuclei were stained with DAPI before observation under a confocal microscope (Zeiss).

To examine the levels of apoptosis in the tissues, terminal deoxynucleotidyl transferase-mediated dNTP nick end-labeling (TUNEL) staining was performed. After anti-tumor therapy, tumors were isolated, fixed with 4% paraformaldehyde, and frozen. Tissue sections were prepared and examined using the ApopTag Red *In situ* Apoptosis Detection kit (Merck), which is based on the TUNEL staining. Nuclei were counterstained with DAPI. Stained tissue samples were observed under a confocal microscope (Zeiss).

### Statistical analysis

Statistical significance was determined using a one-way ANOVA followed by Dunnett's post hoc test for multiple comparisons, or using Student's *t*-test for comparisons between two groups. P values of < 0.05 were considered statistically significant.

## Results

### Screening of peptides that bind to CD44v6 using a phage-displayed random peptide library

**Figure 1:**
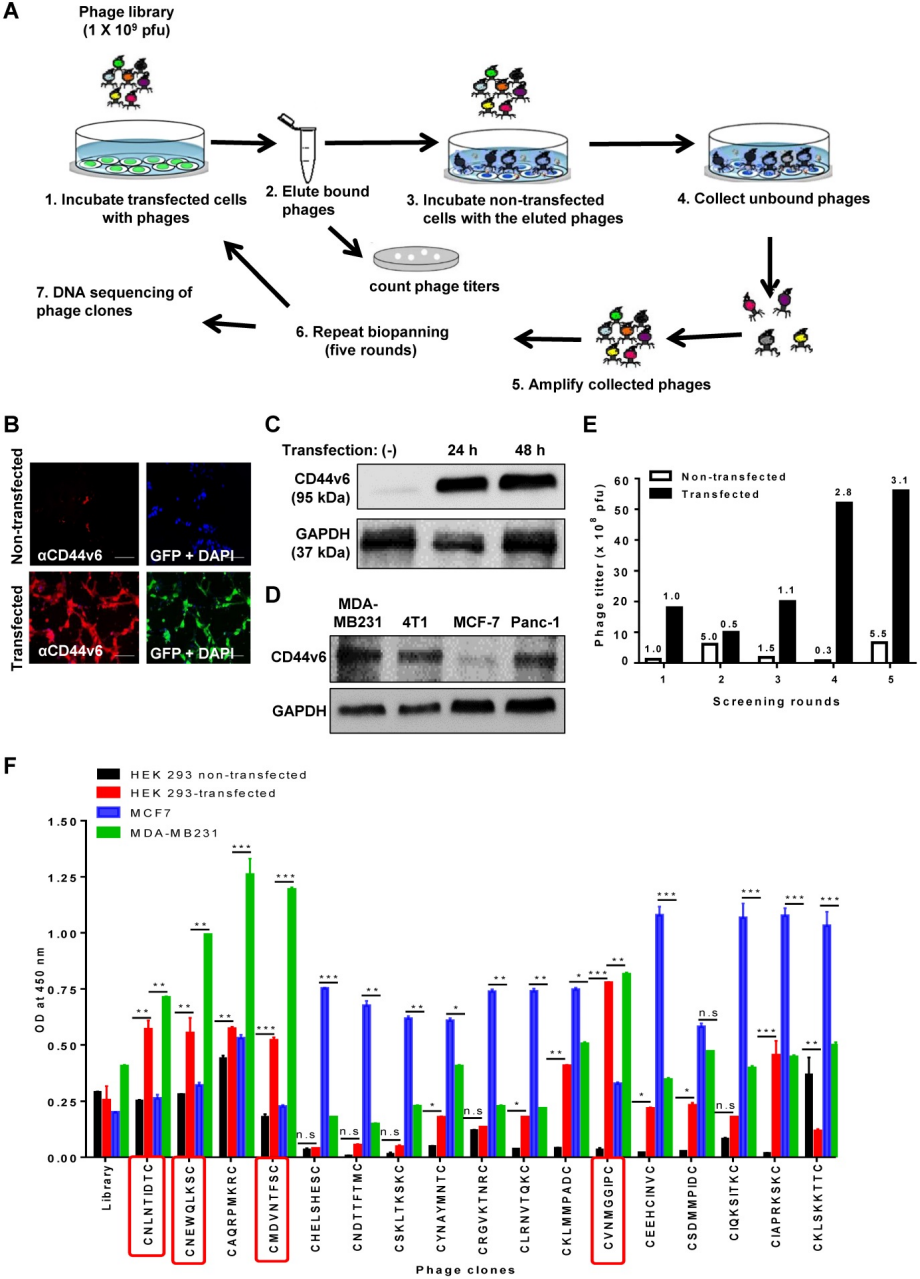
** Screening of a phage peptide library and phage cell-binding ELISA to identify CD44v6-binding peptides.** (A) Experimental schemes for phage peptide library screening. (B) Immunofluorescence staining of CD44v6 with an anti-CD44v6 antibody (red) in HEK 293T cells transfected or not with a GFP-tagged CD44v6 expression vector (green). DAPI was used for nuclear staining (blue). Scale bars = 40 µm. (C) Western blotting analysis of CD44v6 expression in non-transfected (-) and transfected HEK 293T cells at 24 and 48 h after transfection. (D) Western blotting analysis of CD44v6 expression in tumor cells. (E) Enrichment of phage titers during screening rounds. After each round, the phage titers (plaque-forming units; pfu) were measured by plaque assays. Numbers represent the fold ratios relative to the first round. (F) The phage cell-binding ELISA of individual phage clones was performed using HEK 293T cells transfected or not with a CD44v6 expression vector, MDA-MB231 cells, and MCF7 cells. *, *P* < 0.05; **, *P* < 0.01; ***, *P* < 0.001; n.s, not significant by one-way ANOVA.

To select CD44v6-binding peptides, we screened a phage library of hydrophobic amino acid-enriched CX7C random peptides for phages that bound selectively to HEK 293T cells transfected with the GFP tagged-CD44v6 expression vector and not to non-transfected cells (Figure 1A). An immunofluorescence analysis demonstrated that transfected cells expressed high levels of CD44v6 and GFP when compared to non-transfected cells (Figure 1B). Also, Western blotting analysis demonstrated the expression of CD44v6 in transfected HEK 293T cells (Figure 1C) and higher basal levels of CD44v6 expression in MDA-MB231, 4T1, and Panc-1 cells than those in MCF7 cells (Figure 1D). After screening, the phage titers obtained from transfected cells at the fifth round increased by approximately three folds relative to those in the first round, whereas the phage titers obtained from non-transfected cells were not enriched (Figure 1E). Seventy phage clones were selected randomly during the fourth and fifth rounds, and the peptide-coding DNA inserts of these clones were sequenced. Among them, 17 phage clones displaying a peptide with the CX7C pattern were selected for further study (Table S1). Subsequently, a phage cell-binding ELISA revealed that phage clones displaying the peptide sequences CNLNTIDTC, CNEWQLKSC, CMDVNTFSC, and CVNMGGIPC bound to CD44v6-transfected HEK 293T cells and CD44v6-high MDA-MB231 cells at higher levels than to non-transfected HEK 293T cells and CD44v6-low MCF7 cells, respectively (Figure 1F).

**Figure 2:**
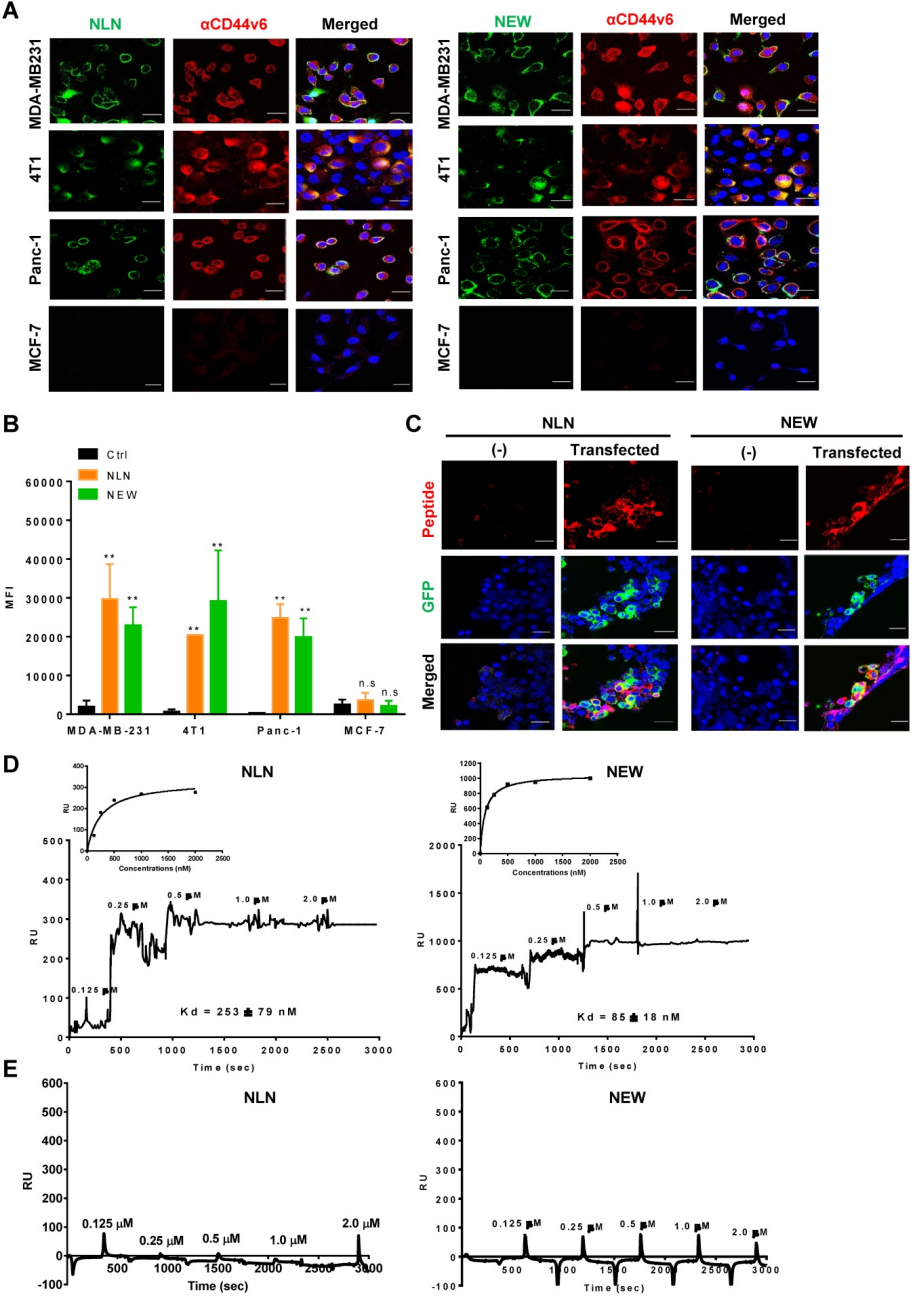
** NLN and NEW bind selectively to CD44v6-overexpressing cells and the CD44v6 protein**. (A) Cellular binding of FITC-labeled NLN and NEW (green, 25 µM) and staining of CD44v6 (red) in CD44v6-high MDA-MB231, 4T1, and Panc-1 cells and CD44v6-low MCF7 cells. Nuclei were stained with DAPI (blue). Scale bars = 20 µm. (B) Mean fluorescence intensities (MFIs) of FITC-labeled NLN or NEW (25 µM) bound to MDA-MB231, 4T1, Panc-1 and MCF7 cells. Data represent the mean MFIs ± standard errors (S.E.) of peptide-bound cells from three separate experiments. **, *P* < 0.01; n.s, not significant compared with the control peptide (Ctrl) by one-way ANOVA. (C) Cellular binding of TAMRA-labeled NLN and NEW (red, 25 µM) and expression of CD44v6 (green) in HEK 293T cells transfected or not with a GFP-tagged CD44v6 expression vector. Nuclei were stained with DAPI (blue). Scale bars = 20 µm. (D-E) SPR analysis of the binding affinity (K_D_ value) of NLN and NEW to recombinant CD44v6-Fc (D) and CD44-Fc (E) proteins. Inset are a binding plot for the peptides. RU, resonance unit.

Next, we examined the binding of synthetic CNLNTIDTC, CNEWQLKSC, CMDVNTFSC, and CVNMGGIPC peptides to CD44v6-expressing cells. Among them, CNLNTIDTC (named NLN) and CNEWQLKSC (named NEW) bound selectively to CD44v6-high cells, such as MDA-MB231, 4T1, and Panc-1 cells, rather than CD44v6-low cells such as MCF7 cells as determined by immunofluorescence microscopy (Figure 2A) and flow cytometry analyses (Figure 2B). Both peptides also preferentially bound to transfected CD44v6-expressing HEK 293T cells relative to non-transfected cells (Figure 2C). Therefore, we chose NLN and NEW as candidates for further studies. Saturation binding assays showed that the K_D_ values of NLN binding to MDA-MB231, 4T1, Panc-1, and MCF7 cells were 12 ± 5, 17 ± 7, 8 ± 5, and 300 ± 250 µM, respectively, and those of NEW binding to the cells were 18 ± 6, 29 ± 16, 7 ± 3 , and 122 ± 115 µM, respectively (Figure S2A).

To measure the binding affinity (K_D_ value) of NLN and NEW to the CD44v6 protein, SPR analysis was performed using recombinant human CD44v6-Fc and CD44-Fc proteins and streptavidin chips coated with biotin-conjugated NLN and NEW. The K_D_ values of NLN and NEW binding to the CD44v6 protein were 253 ± 79 and 85 ± 18 nM, respectively (Figure 2D). In contrast, both peptides did not bind to the CD44 protein (Figure 2E). These findings indicate that NLN and NEW selectively bind to CD44v6 rather than CD44. In addition, NLN and NEW preferentially bound to the recombinant CD44v6-Fc protein coated on plates compared with CD44-Fc protein and albumin as controls (Figure S2B).

### Cellular binding of NLN and NEW is mediated by CD44v6

**Figure 3:**
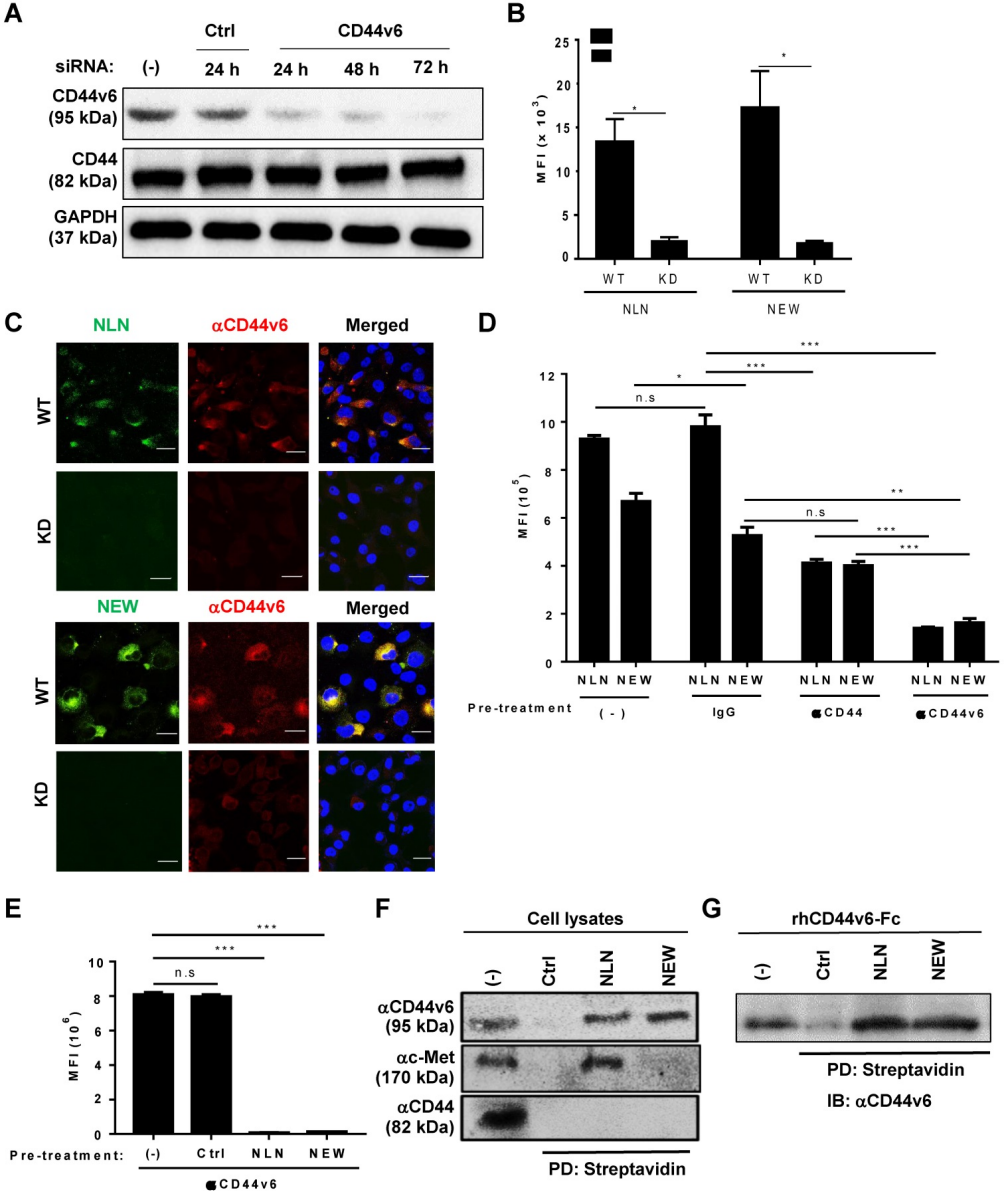
** Cellular binding of NLN and NEW is mediated by CD44v6.** (A) Western blotting analysis of CD44v6 and CD44 levels in MDA-MB231 cells after the knockdown (KD) of CD44v6 gene expression for 24, 48, and 72 h in cells treated with CD44v6 siRNA or control siRNA. GAPDH is used as a control protein. (B) The MFI of wild-type (WT) and CD44v6 KD MDA-MB231 cells bound to FITC-labeled NLN and NEW (25 µM). Data are shown as mean MFIs ± S.E. of peptide-bound cells from three separate experiments. *, *P* < 0.05 by Student's *t*-test. (C) Confocal microscopic images of WT and KD MDA-MB231 cells bound with FITC-labeled NLN and NEW (green, 25 µM) and stained with an anti-CD44v6 antibody (red) and DAPI (blue). Scale bars = 20 µm. (D) Competitive binding of FITC-labeled NLN and NEW (10 µM) following pre-treatment with anti-CD44v6 and anti-CD44 antibodies and IgG control in MDA-MB231 cells. The mean MFIs ± S.E. of peptide-bound cells from three separate experiments are shown. *, *P* < 0.05; **,* P* < 0.01; ***,* P* < 0.001; n.s, not significant by one-way ANOVA. (E) Competitive binding of an anti-CD44v6 antibody following pre-treatment with NLN and NEW (50 µM) in MDA-MB231 cells. ***,* P* < 0.001; n.s, not significant compared with untreated control by one-way ANOVA. (F) Pull-down assay of CD44v6 using biotin-labeled NLN and NEW and streptavidin beads, followed by immunoblotting with anti-CD44v6, anti-c-Met, and anti-CD44 antibodies. (G) Pull-down assays of the recombinant human CD44v6-Fc protein using biotin-labeled NLN and NEW and streptavidin followed by immunoblotting (IB) with anti-CD44v6 antibody. Ctrl, control peptide.

To examine whether CD44v6 mediated the cellular binding of NLN and NEW, we performed CD44v6 gene expression knockdown, competitive binding, and cell lysate pull-down experiments. The transfection of a CD44v6 siRNA into MDA-MB231 cells knocked down CD44v6 gene expression and reduced CD44v6 protein levels at 24, 48, and 72 h after transfection, without affecting the levels of CD44 and GAPDH as controls (Figure 3A). Accordingly, the cellular binding of NLN and NEW to the CD44v6-knockdown cells decreased by 6-7-fold compared with wild-type cells as determined by flow cytometry (Figure 3B) and immunofluorescence microscopy analyses (Figure 3C). The pre-treatment of MDA-MB231 cells with an anti-CD44v6 antibody significantly inhibited the subsequent binding of NLN and NEW to the cells, while an anti-CD44 antibody and an IgG control did not (Figure 3D); however, pre-treatment of the cells with the anti-CD44 antibody reduced the subsequent binding of NLN compared with the IgG control (Figure 3D). In addition, pre-treatment of MDA-MB231 cells with NLN and NEW, but not with control peptide, inhibited the subsequent staining of CD44v6 with the anti-CD44v6 antibody (Figure 3E). Furthermore, the incubation of cell lysates with biotinylated NLN and NEW, but not control peptides, followed by streptavidin beads selectively pulled down CD44v6 but not CD44, whereas NLN pulled down both c-Met and CD44v6 (Figure 3F). Incubation of the recombinant human CD44v6-Fc protein with biotinylated NLN and NEW, but not control peptides, also pulled down the protein (Figure 3G). Collectively, these results indicate that NLN and NEW bind to CD44v6-epxressing cells specifically through CD44v6.

### NLN and NEW inhibit HGF-induced c-Met internalization, c-Met phosphorylation, and cell migration and invasion of tumor cells

**Figure 4:**
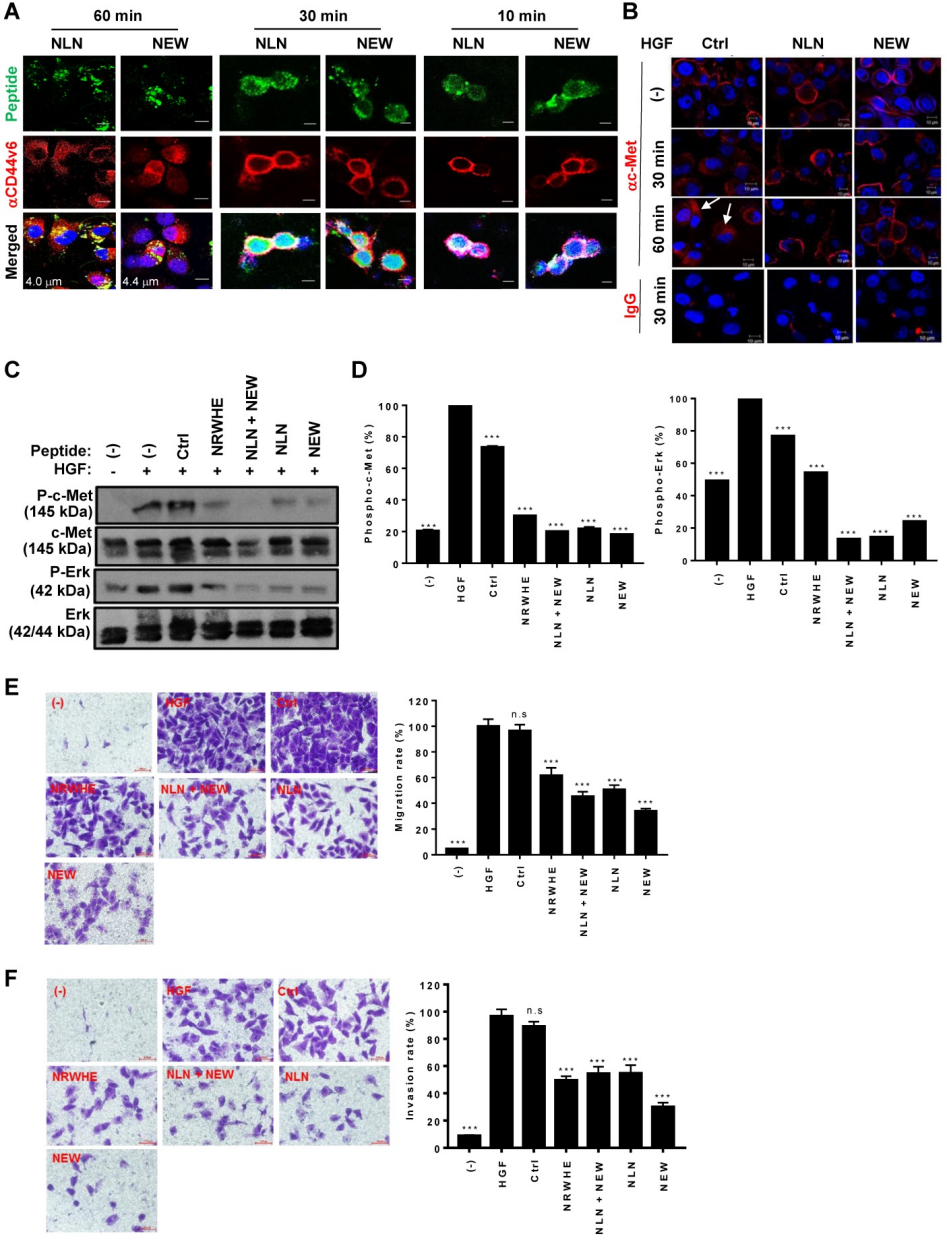
** NLN and NEW inhibit HGF-induced c-Met internalization, c-Met phosphorylation, and cell migration and invasion in MDA-MB231 breast tumor cells.** (A) A confocal microscopic Z-section analysis of the internalization of NLN and NEW, and CD44v6 into MDA-MB231 cells. Cells were incubated with FITC-labeled peptides (green, 10 µM) at 37 °C for 10, 30, and 60 min and stained with an anti-CD44v6 antibody (red). Nuclei were stained with DAPI (blue), and images were merged. Numbers in the merged images indicate the distances from the cell surfaces in µm. Scale bars = 10 µm. (B) Confocal microscopic analysis of c-Met (red) in MDA-MB231 cells after pre-treatment with NLN and NEW (10 µM) at 37 °C for 10 min and subsequent treatment with 25 ng/mL HGF for 30 or 60 min. Arrows indicate cytoplasmic c-Met. Nuclei were stained with DAPI (blue). Scale bars = 10 µm. (C) Western blotting analysis of c-Met and Erk phosphorylation in MDA-MB231 cells pre-treated or not with NLN and NEW (20 µM) for 10 min and subsequently treated with 25 ng/mL HGF for 10 min. (D) Phosphorylated c-Met and Erk protein levels normalized by c-Met and Erk total protein levels in MDA-MB231 cells. (E-F) Transwell migration (E) and invasion (F) assays of MDA-MB231 cells pre-treated or not with NLN and NEW (20 µM) for 10 min and subsequently treated with 25 ng/mL HGF for 10 min, followed by incubation for 24 h. Scale bars = 20 µm. Graphs (right panels) represent the quantification of the cell numbers in ten randomly selected fields. Data are shown as the means ± S.E. of three independent experiment. ***,* P* < 0.001; n.s, not significant compared with HGF by one-way ANOVA.

We examined the effects of NLN and NEW on CD44v6 internalization and HGF-induced c-Met internalization. When MDA-MB231 cells were incubated with NLN and NEW for 60 min, both FITC-labeled peptides and antibody-stained CD44v6 were observed in the cytoplasm of the cells (Figure 4A), indicating the internalization of CD44v6-bound NLN and NEW. In addition, both the peptides and antibody-stained CD44v6 were observed at the cell surface after incubation for 10 min, and a part of the peptides were internalized while CD44v6 was observed at the cell surface after incubation for 30 min (Figure 4A), indicating a time-dependent internalization of the peptides and CD44v6. As expected, treatment with HGF for 30 or 60 min efficiently induced the internalization of c-Met into the cytoplasm of MDA-MB231 cells (Figure 4B). However, pre-treatment with either NLN and NEW, but not the control peptide, before HGF treatment subsequently inhibited the HGF-induced internalization of c-Met (Figure 4B).

As the interaction between CD44v6 and c-Met is important for c-Met downstream signaling and cell migration, we examined the effects of NLN and NEW on HGF-induced c-Met and Erk phosphorylation and cell migration. Pre-treatment with NLN and NEW, either alone or in combination, inhibited the HGF-induced phosphorylation of c-Met and Erk in MDA-MB231 (Figure 4C-D) and 4T1 cells (Figure S3A-B). In addition, pre-treatment with NLN and NEW, but not control peptide, inhibited the HGF-induced cell migration and invasion in MDA-MB231 cell lines (Figure 4E-F) and 4T1 cells in (Figure S3C-D). These results were comparable to those obtained using NRWHE, a previously reported CD44v6-derived peptide 24.

### NLN and NEW home to CD44v6-expressing tumors *in vivo*

**Figure 5:**
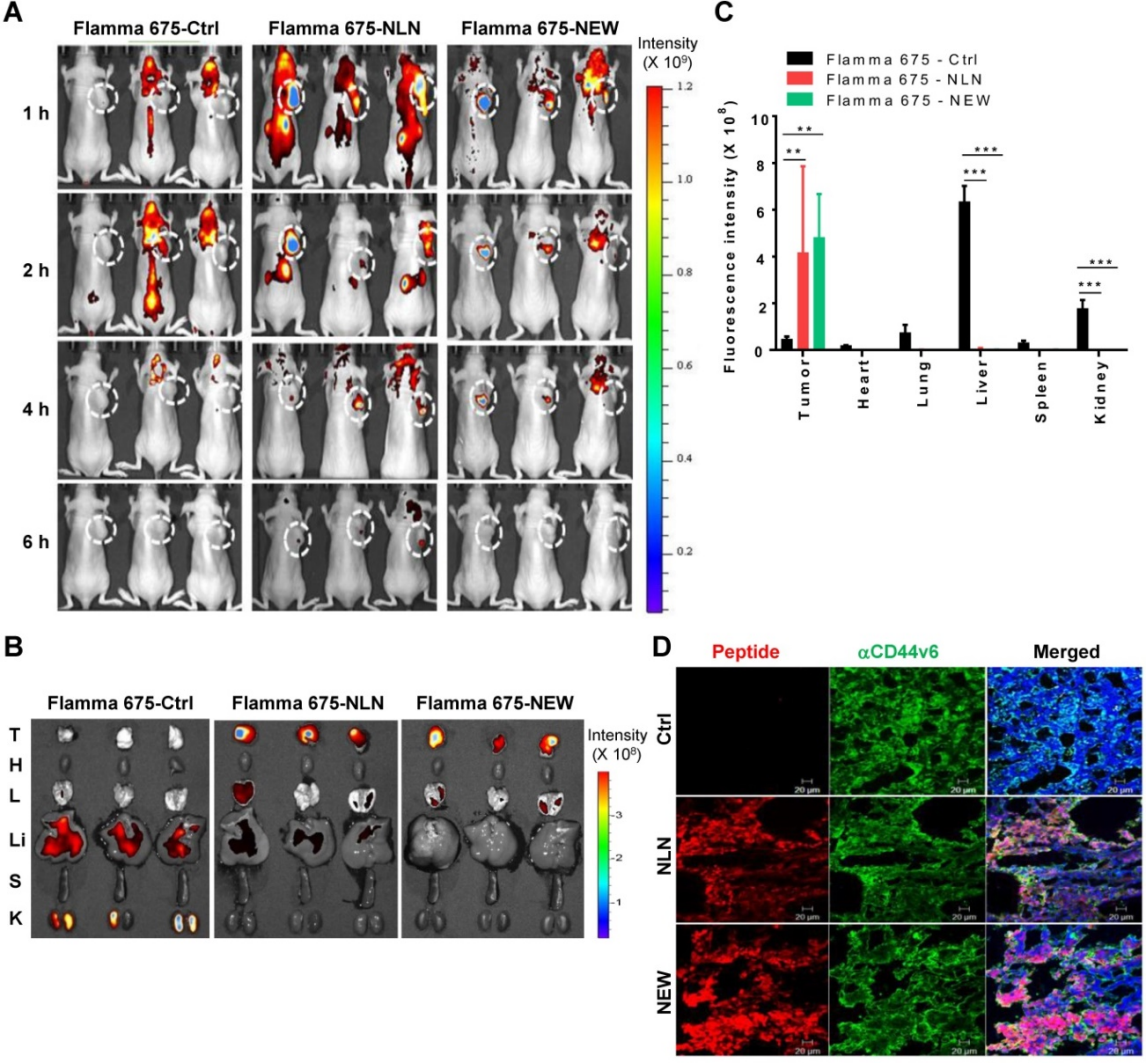
***In vivo* whole-body fluorescence imaging of NLN and NEW homing to MDA-MB231 breast tumor in mice.** (A) *In vivo* whole-body fluorescence imaging of the homing of Flamma 675 NIR dye-labeled NLN, NEW, or control peptide to tumors at 1-6 h after injection into BALB/c nude mice. Dotted circles represent the tumor region. The scale bar indicates the normalized fluorescent intensity. (B) *Ex vivo* imaging of the accumulation of Flamma 675 NIR dye-labeled NLN, NEW, or control peptide in the tumors and other organs isolated from mice 6 h after peptide injection. The scale bar indicates the normalized fluorescence intensity. T, tumor; H, heart; L, lung; Li, liver; S, spleen; K, kidney. (C) Quantification of the *ex vivo* fluorescence intensities in the tumor and organs. Data are shown as the means ± S.E. (*n* = 3/group). **,* P* < 0.01; ***,* P* < 0.001 compared with the control peptide by one-way ANOVA. (D) Co-localization of CD44v6 (green) with NLN and NEW (red) in tumor tissue sections. Nuclei were counter-stained with DAPI (blue). Scale bars = 20 µm. Ctrl, control peptide.

To examine the homing of NLN and NEW to tumors, Flamma 675 NIR fluorescence dye-labeled peptides were injected intravenously into BALB/c nude mice bearing MDA-MB231 subcutaneous tumors, and the mice were subjected to *in vivo* whole-body fluorescence imaging. Fluorescence signals indicating the homing of NLN and NEW to tumor areas were detected as early as 1 h and were higher than those associated with the control peptide until 4 h and declined by 6 h after peptide injection (Figure 5A). The *ex vivo* fluorescence imaging of isolated tumors and organs at 6 h post-injection further demonstrated higher levels of NLN and NEW accumulation in tumors than in control organs, including the livers and lungs, whereas higher accumulation of the control peptide was observed in the livers and kidneys than in tumors (Figure 5B-C). An immunohistochemical analysis demonstrated that NLN and NEW accumulated in tumor tissues, where it co-localized with CD44v6 (Figure 5D). These results suggest that NLN and NEW are efficient imaging probes that are taken up rapidly by CD44v6-expressing tumor tissues.

### NLN and NEW inhibit the metastasis of malignant breast tumors

**Figure 6:**
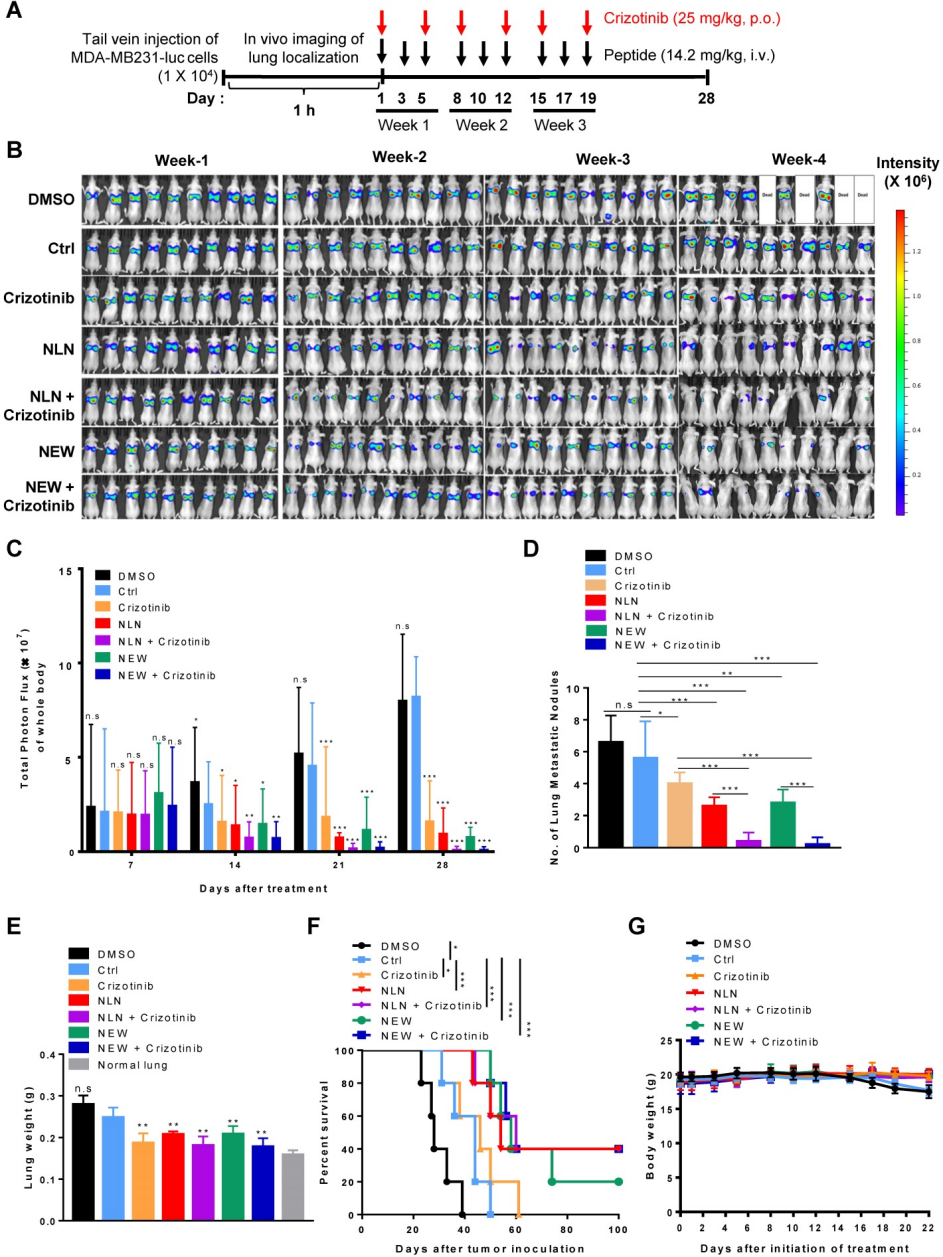
** NLN and NEW inhibit tumor metastasis to the lung in MDA-MB231 experimental breast tumor metastasis model.** (A) Treatment protocols. MDA-MB231-luc cells were injected intravenously into BALB/c nude mice. At 1 h post-injection, the mice received intravenously injected NLN, NEW, or control peptide (14.2 mg/kg body weight, black arrows, thrice weekly for 3 weeks) and orally administered crizotinib (25 mg/kg body weight, red arrows, twice weekly for 3 weeks). (B) *In vivo* whole-body luminescence imaging and monitoring of metastatic tumor growth in the lungs of mice treated with either NLN or NEW and crizotinib alone or in combination. (C) Quantification of the total photon flux (number of photons/second) in the whole body. (D) Number of metastatic tumor nodules in the lungs. (E) Lung weights. (F) Survival rates. (G) Body weights. Data are shown as the means ± S.E. (*n* = 10/group). *, *P* < 0.05; **,* P* < 0.01; ***,* P* < 0.001 compared with control peptide by one-way ANOVA. Ctrl, control peptide.

To mimic the metastasis of breast cancer to the lung, MDA-MB231-luc cells, which express luciferase, were injected intravenously into BALB/c nude mice as an experimental tumor metastasis model 30, 31. A whole-body bioluminescence imaging showed the localization of tumor cells at the lungs within 1 h after injection, and mice were classified into groups based on the initial luminescence. NLN and NEW were administered intravenously into mice at 1 h post-tumor cell injection, which mimics an early stage of metastasis, and administered thrice weekly thereafter for 3 weeks (Figure 6A). Treatment with either NLN or NEW decreased the luminescence signals and total photon flux in the bodies of mice, as determined by *in vivo* whole-body luminescence imaging (Figure 6B-C, respectively), and reduced both the numbers of tumor nodules (Figure 6D) and weights of the isolated lungs (Figure 6E). In addition, treatment with either NLN or NEW increased the survival rate (Figure 6F). These findings suggest that metastatic tumor growth in the lungs was inhibited by treatment with either NLN or NEW.

The oral administration of crizotinib, a Food and Drug Administration-approved small molecule c-Met inhibitor, also inhibited the growth of MDA-MB231-luc metastases in the lungs and increased the survival rate (Figure 6B-F). Treatment with either NLN and NEW in combination with crizotinib (25 mg/kg body weight) more efficiently reduced the number of tumor nodules in the isolated lungs and increased the survival rate than did a single treatment with either peptide or crizotinib alone (Figure 6B-F). None of the treatments significantly affected the body weights (Figure 6G). The liver and kidney functional marker levels were within or close to the normal ranges after treatment of BALB/c nude mice bearing MDA-MB231 tumor with either NLN and NEW alone or in combination with crizotinib (Figure S4). However, NLN significantly increased the serum alanine transferase levels above the normal ranges (Figure S4D).

To generate a spontaneous tumor metastasis model, 4T1-luc cells were implanted orthotopically into the mammary fat pads of BALB/c mice (Figure S5A). The intravenous administration of either NLN or NEW decreased the luminescence signals and total photon flux emitted from the whole body, as demonstrated by *in vivo* whole-body bioluminescence imaging (Figure S5B and C, respectively), and also decreased the volumes (Figure S5D) and weights of isolated primary tumors (Figure S5E), indicating that the peptides inhibited primary tumor growth. Moreover, treatment with either NLN or NEW inhibited metastatic tumor growth in the lungs, as indicated by a reduced number of lung tumor nodules (Figure S5F) relative to those in the saline and control peptide-treated groups. Either NLN or NEW treatment increased the survival rate (Figure S5G) and had little effect on the body weight (Figure S5H). Combined treatment with either NLN or NEW and crizotinib (25 mg/kg body weight) inhibited primary tumor growth and lung metastasis and increased survival rates more efficiently than a single treatment with NLN, NEW, or crizotinib (Figure S5). The hematologic parameters and liver and kidney functional marker levels were not significantly changed or remained within normal ranges after treatment of BALB/c mice bearing 4T1 tumor with either NLN or NEW alone or in combination with crizotinib compared with control peptide (Figure S6). However, the monocyte population was decreased below the normal ranges after treatment with NLN (Figure S6G).

Next, we examined whether NLN or NEW in combination with a lower dose of crizotinib can induce the same therapeutic effect of a higher dose of crizotinib alone. The combined treatment of either NLN or NEW and crizotinib (12.5 mg/kg body weight) inhibited tumor growth and lung metastasis and enhanced mouse survival rate and TUNEL-stained apoptosis levels more efficiently than crizotinib alone (25 mg/kg body weight) (Figure S7).

### NLN and NEW deliver pro-apoptotic peptide to tumors and inhibit primary tumor growth and metastasis

**Figure 7:**
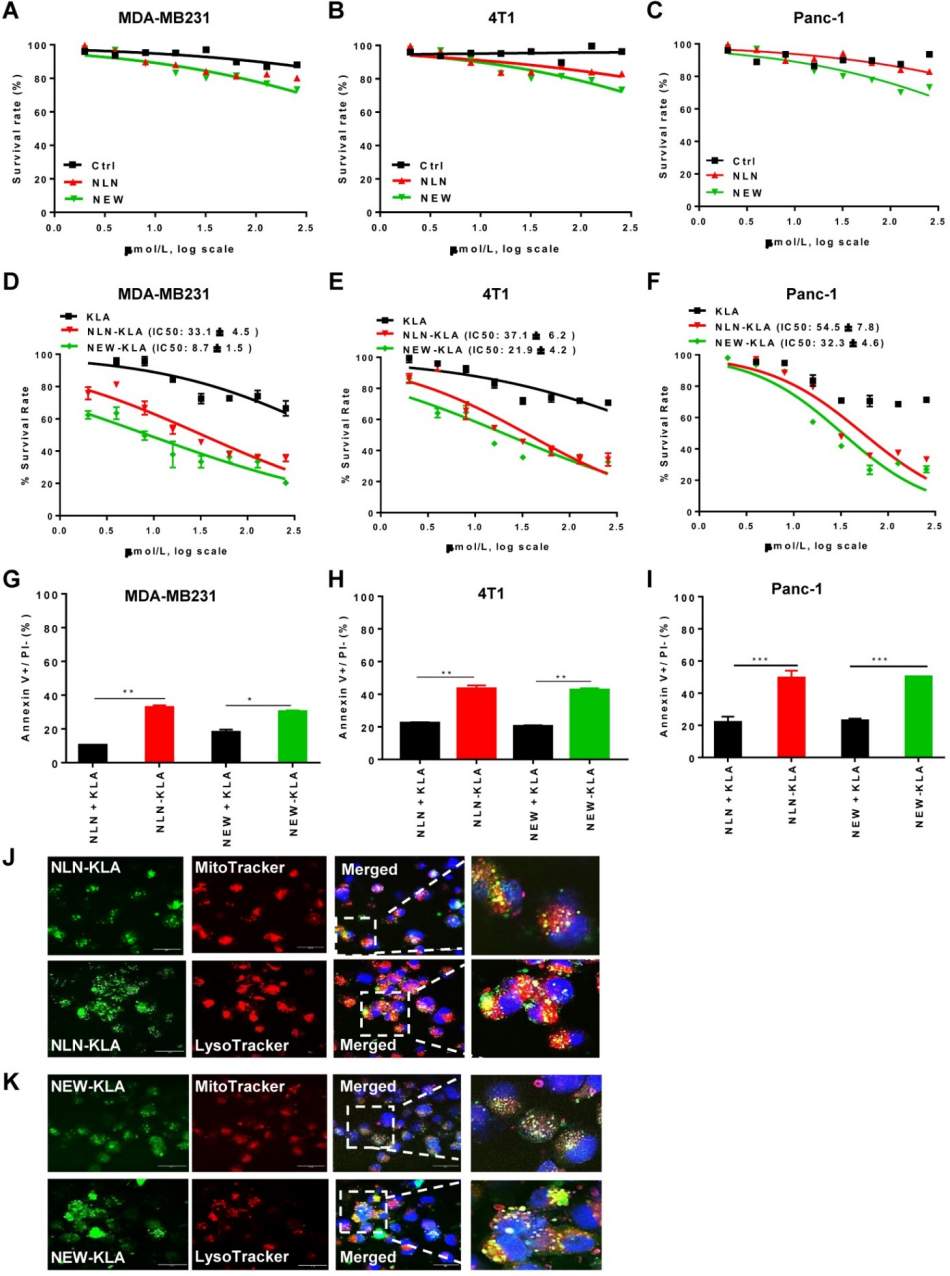
** NLN-KLA and NEW-KLA exert cytotoxicity and induce apoptosis in tumor cells.** (A-C) MDA-MB231 (A), 4T1 (B), and Panc-1 cells (C) were treated with NLN, NEW, or control peptide (Ctrl) at the indicated concentrations for 24 h, and cytotoxicity was determined. (D-F) MDA-MB231 (D), 4T1 (E), and Panc-1 cells (F) were treated with NLN-KLA, NEW-KLA, or KLA at the indicated concentrations for 24 h, and cytotoxicity was determined. Data are mean ± S.E. of three independent experiments performed in a triplicate. (G-I) Percent apoptotic cells (annexin V+/propidium iodide-) in MDA-MB231, 4T1 and Panc-1 cells was determined after treatment with NLN-KLA, NEW-KLA, or combined treatment of either NLN or NEW and KLA. Data are mean ± S.E. of three independent experiments performed in a triplicate. *, *P* < 0.05; **,* P* < 0.01; ***,* P* < 0.001; n.s, not significant by one-way ANOVA. (J-K) Intracellular trafficking of NLN-KLA and NEW-KLA. For staining of mitochondria and lysosomes, MDA-MB231 cells were incubated with NLN-KLA and NEW-KLA (green) for 2 h and 4 h, and then incubated with MitoTracker and Lysotracker dye (red), respectively. Nuclei were counter-stained with DAPI (blue). Scale bars = 20 µm.

Treatment with either NLN or NEW reduced cell survival only by 20% even at concentrations of ≥100 µM in cultures of MDA-MB231, 4T1, and Panc-1 cells (Figure 7A-C, respectively), suggesting that the CD44v6-binding peptides *per se* are weakly cytotoxic. To directly kill tumor cells, we exploited KLAKLAKKLAKLAK (named KLA), a pro-apoptotic peptide, and synthesized hybrid peptides consisting of either NLN or NEW and KLA (named NLN-KLA and NEW-KLA, respectively). NLN-KLA exerted cytotoxicity to MDA-MB231, 4T1, and Panc-1 cells, resulting in IC50 values of 33.1 ± 4.5 µM, 37.1 ± 6.2 µM, and 54.5 ± 7.8 µM, respectively (Figure 7D-F). Also, NEW-KLA showed cytotoxicity to MDA-MB231, 4T1, and Panc-1 cells, resulting in IC50 values of 8.7 ± 1.5 µM, 21.9 ± 4.2 µM, and 32.3 ± 4.6 µM, respectively (Figure 7D-F). Moreover, NLN-KLA and NEW-KLA induced apoptosis in MDA-MB231, 4T1, and Panc-1 cells more efficiently than the combined treatment of either NLN or NEW and KLA did (Figure 7G-I). Confocal microscopic analysis demonstrated that NLN-KLA and NEW-KLA closely co-localized with mitochondria and lysosomes in the cytoplasm at 2-4 h after incubation with MDA-MB231 cells (Figure 7J-K), suggesting that a portion of the peptides escaped from the endosomes/lysosomes after internalization and targeted mitochondria, whereas a portion of the peptides were trapped in the lysosomes.

**Figure 8:**
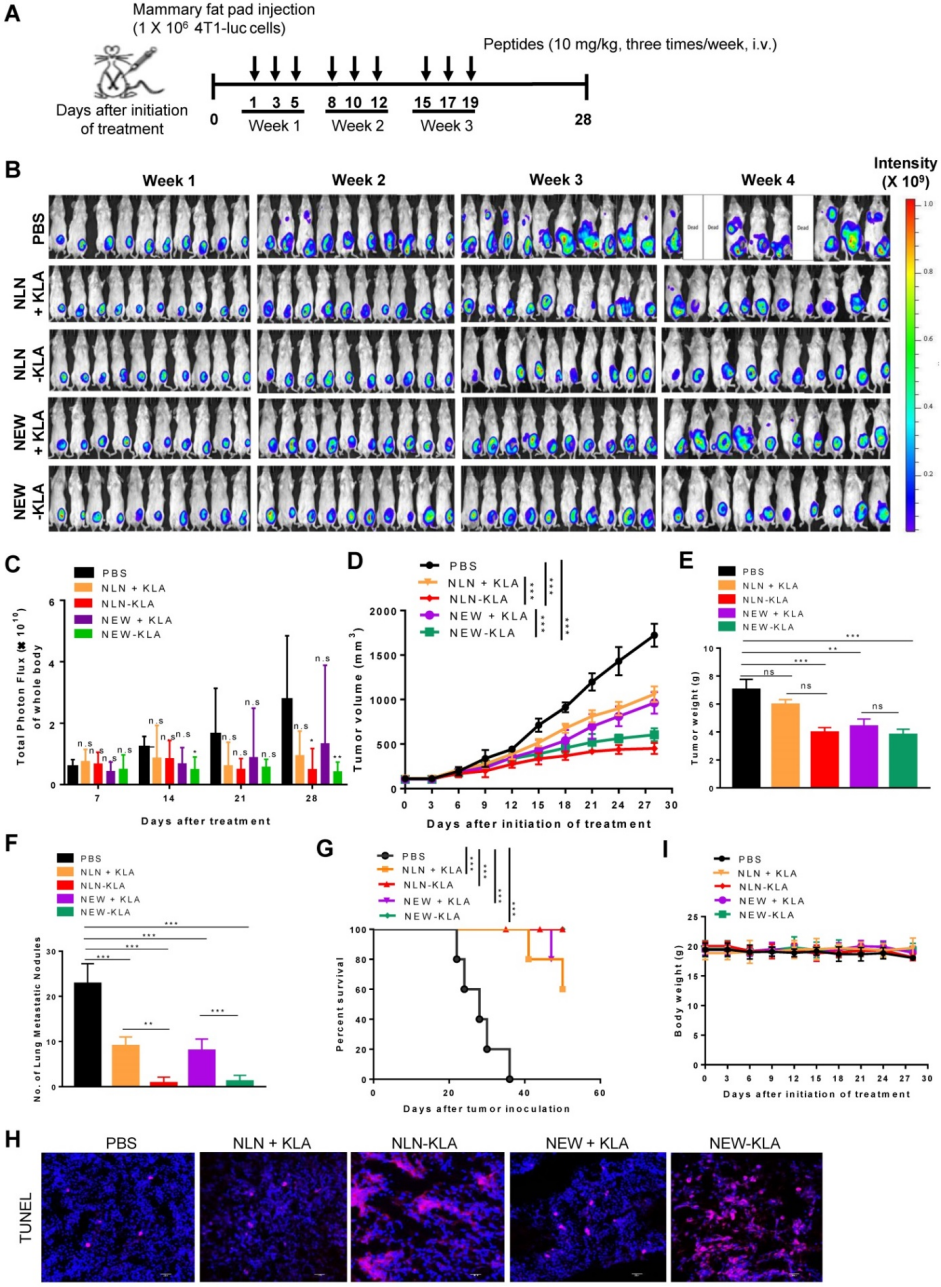
** NLN-KLA and NEW-KLA inhibit the primary tumor growth and metastasis in 4T1 spontaneous breast tumor metastasis model.** (A) Treatment protocols. NLN-KLA, NEW-KLA, combination of NLN and KLA (NLN + KLA), or combination of NEW and KLA (NEW + KLA) was intravenously injected into BALB/c mice bearing 4T1 tumor at mammary fat pads at the indicated time points (10 mg/kg body weight, thrice per week for three weeks). (B) Whole-body bioluminescence imaging to monitor tumor progression. (C) Quantification of the total photon flux (number of photons/second) in the whole body. (D) Tumor volumes. (E) Tumor weights. (F) Numbers of metastatic tumor nodules in the lungs. (G) Percent survival. (H) TUNEL staining (red) in primary tumor tissues. Nuclei were stained with DAPI (blue). Scale bars = 20 µm. (I) Body weights. Data are mean ± S.E. (*n* = 10 per group). *, *P* < 0.05; **,* P* < 0.01; ***,* P* < 0.001; n.s, not significant compared with phosphate-buffered saline (PBS) by one-way ANOVA.

To examine whether NLN-KLA and NEW-KLA inhibit tumor growth and metastasis, the peptides were intravenously administered into BALB/c mice bearing 4T1-luc breast tumor implanted orthotopically into the mammary fat pads (Figure 8A). Either NLN-KLA or NEW-KLA decreased the luminescence signals and total photon flux emitted from the whole body, as demonstrated by *in vivo* whole-body bioluminescence imaging (Figure 8B-C, respectively), reduced tumor volume (Figure 8D), tumor weight (Figure 8E), and number of metastatic tumor nodules (Figure 8F), while increasing percent survival (Figure 8G) and TUNEL-positive apoptotic cells (Fig. 8H), more efficiently than the combined treatment of either NLN or NEW and KLA. Body weights were not changed by the treatments (Figure 8I). Moreover, the inhibition of tumor growth and metastasis by either NLN-KLA or NEW-KLA were more efficient than those by the combined treatment of either NLN or NEW and crizotinib (Figure S5). The hematologic parameters and liver and kidney functional marker levels were within or close to the normal ranges after treatment of BALB/c mice bearing 4T1 tumor with either NLN-KLA or NEW-KLA (Figure S8). However, the white blood cell, lymphocyte, and monocyte population were below the normal ranges, while lymphocyte and monocyte population were increased to the normal ranges after the combined treatment with NEW and KLA (Figure S8F-G, respectively). As another model of spontaneous tumor metastasis, the peptides were intravenously administered into mice bearing subcutaneous Panc-1 pancreatic tumor (Figure S9A). Either NLN-KLA or NEW-KLA inhibited tumor growth and metastasis to the lungs and increased percent survival and TUNEL-positive apoptotic cells in tumor tissues more efficiently than the combined treatment of either NLN or NEW and KLA (Figure S9B-G).

## Discussion

In this study, we identified two CD44v6-binding peptides, NLN and NEW, through a phage-displayed peptide library screening analysis and determined that the peptides not only bound selectively to CD44v6, but also inhibited the interaction between CD44v6 and c-Met. Both peptides bound to CD44v6-high cells and pulled down CD44v6 from cell lysates. The cellular binding of these peptides was reduced by siRNA-mediated CD44v6 knockdown or by competitive binding with an anti-CD44v6 antibody. These findings demonstrate that NLN and NEW bind to CD44v6-expressing cells specifically through CD44v6. Once bound, these peptides induced the internalization of CD44v6 into the cytoplasm and inhibited the HGF-induced internalization of c-Met. Treatment of human and mouse breast tumor cells with the CD44v6-binding peptides inhibited the HGF-induced phosphorylation of c-Met and downstream signaling factors such as Erk and subsequently inhibited cell migration and invasion. The CD44v6-binding peptides homed to CD44v6-expressing tumors *in vivo* and could be used to detect the tumors via imaging. NLN and NEW inhibited primary tumor growth of mouse syngeneic breast tumor. Moreover, systemic NLN and NEW administration inhibited the metastasis of human and mouse breast cancer cells to the lungs in mice in experimental and spontaneous metastasis model, respectively.

The c-Met inhibitor crizotinib has been used widely as a cancer therapeutic agent. Crizotinib also inhibits anaplastic lymphoma kinase (ALK) and has shown remarkable therapeutic efficacy in patients with ALK-positive non-small cell lung cancer 29, 32. Moreover, combined treatment with crizotinib and vemurafenib, a BRAF inhibitor, yielded beneficial therapeutic effects in patients with colorectal cancer 33, 34. However, the use of crizotinib is hindered by systemic side effects, including hepatotoxicity 29. In this study, combined treatment with either NLN or NEW and crizotinib at a lower dose (12.5 mg/kg body weight) inhibited tumor growth and metastasis more efficiently than treatment with crizotinib alone (25 mg/kg body weight). Moreover, in mice, liver and kidney functional marker levels remained within the normal ranges after treatments with the CD44v6-binding peptides in combination with crizotinib. Therefore, it is reasonable to hypothesize that the addition of either NLN or NEW would enhance the therapeutic index of crizotinib while reducing the required dosage and systemic side effects.

The CD44v6-derived peptides, KEQWFGNRWHEGYR, QETWFQNGWQGKNP, and KEKWFENEWQGKNP, were identified by a mutational analysis of the v6 domains of human, mouse, and rat CD44v6, respectively (the five amino acids essential for activity are underlined) 24, 28. The CD44v6-derived peptides inhibit pancreatic tumor growth and metastasis in mice more efficiently than crizotinib and pazopanib, a small molecule VEGFR-2 inhibitor 28. As the CD44v6-derived peptides are derived from the endogenous sequence of CD44v6, they inhibit the CD44v6 and c-Met interaction by competing against the v6 domain of CD44v6. We found that the NEWQ sequence of CNEWQLKSC shares homology with the NEWQG peptide derived from the v6 domain of CD44v6. This suggests that, similarly to the NEWQG peptide, NEW interferes with the CD444v6 and c-Met interaction and inhibits breast tumor growth and metastasis presumably by mimicking CD44v6 and competing against the v6 domain. In addition, an NCBI protein database search revealed that the NLN amino acid sequence of CNLNTIDTC shares homology with the ^766^NLN^768^and ^784^NLN^786^ sequences of c-Met isoforms 1 and 2, respectively. NLN peptide does not share homology with a previously reported c-Met-binding peptide, YLFSVHWPPLKA, which was selected from phage-displayed random peptides and was shown to detect c-Met-expressing tumors in mice 35. These findings suggest that NLN binds to CD44v6 and interferes with the interaction between CD44v6 and c-Met presumably by mimicking c-Met.

It has been shown that CD44v6, c-Met, and HGF form a multimeric complex, and antibodies to the v6 region of CD44v6 interfere with such complex formation and subsequently inhibit the phosphorylation of c-Met 8. Similar to the v6 antibodies, the mechanism of action of NLN and NEW would be to interfere with the formation of CD44v6/c-Met/HGF complex and inhibit the c-Met downstream signaling pathways. In support, pre-treatment with NLN and NEW inhibits the internalization and phosphorylation of c-Met as well as cell migration and invasion induced by HGF treatment. In addition, NLN pulled down both c-Met and CD44v6, whereas NEW pulled down only CD44v6, suggesting that NEW may interfere with the interaction between CD44v6 and c-Met more efficiently than NLN. However, it does not seem to result in a significant difference in the behavior of the two peptides.

Unlike to the CD44v6-derived peptides, NEW and NLN were identified by screening a phage-displayed random peptide library for peptides that bind to CD44v6-overexpressing cells. The main advantage of this approach is that the selected peptides may not only block the interaction between CD44v6 and c-Met but also bind to and subsequently induce the internalization of CD44v6 into the cytoplasm acting as a ligand. The latter property enables the peptides to deliver therapeutics or nanoparticles into CD44v6-high tumor cells through the receptor-mediated endocytosis. Moreover, they can be used for the detection of CD44v6-high tumors as a molecular imaging probe. In support of this, NLN and NEW successfully delivered the KLA pro-apoptotic peptide to tumor cells, resulting in the internalization of KLA and induction of tumor cell death. KLA, originally developed as an anti-microbial peptide, induces a damage of mitochondrial membrane and subsequently apoptotic cell death 36. However, KLA does not cross the cell membrane due to highly charged amino acids and needs a targeting moiety that binds to a cell membrane receptor for its internalization 37. Interestingly, the KLA pro-apoptotic peptides guided by either NLN (NLN-KLA) or NEW (NEW-KLA) inhibited tumor growth as well as metastasis in the 4T1 spontaneous tumor metastasis model more efficiently than the combined treatment of each peptide and crizotinib did.

## Conclusion

Collectively, our study results suggest that the NLN and NEW CD44v6-binding peptides are potential metastasis-inhibiting peptide therapeutic agents when administered alone or in combination with crizotinib to patients with CD44v6-high tumors. Also, the results suggest that the NLN and NEW can be used as targeting moieties for the selective delivery of pro-apoptotic peptides and drug-loaded nanoparticles to CD44v6-expressing tumors when labeled on the surfaces of the nanocarriers. These peptides could also be used as imaging probes for the detection of CD44v6-high tumors if conjugated with positron emission tomography radioisotopes such as fluorine-18. Moreover, our study results suggest that NLN-KLA and NEW-KLA are novel therapeutics that targets the mitochondrial membrane of CD44v6-expressing tumor cells.

## Supplementary Material

Supplementary figures.

## Abbreviations

CD44v6: CD44 variant 6
HGF: hepatocyte growth factor
VEGFR-2: vascular endothelial growth factor receptor-2
ATCC: American type culture collection
DMEM: Dulbecco's modified Eagle's medium
RPMI: Roswell park memorial institute medium
FBS: fetal bovine serum
GFP: green fluorescent protein
FITC: fluorescein isothiocyanate
TAMRA: 5-carboxytetramethylrhodamine
NIR: Near-infrared
BSA: bovine serum albumin
DAPI: 4ʹ,6-diamidino-2-phenylindole
siRNA: Small interfering RNA
DMSO: dimethyl sulfoxide
TUNEL: terminal deoxynucleotidyl transferase-mediated dNTP nick end-labeling
ALK: anaplastic lymphoma kinase
