# Supplementary material for: Identification of novel CD44v6-binding peptides that block CD44v6 and deliver a pro-apoptotic peptide to tumors to inhibit tumor growth and metastasis in mice
Source: Theranostics. 2021 Jan 1;11(3):1326–44. doi: 10.7150/thno.50564 (PMC7738880; doi:10.7150/thno.50564)
Supplement: Supplementary file 1 — Supplementary figures. [file thnov11p1326s1.pdf]

Supplementary Information

Supplementary Materials and Methods

Saturation binding assays to determine the cellular binding affinities of peptides

Cells (5×10^3 cells per well in a 96-well plate) were incubated with 1% bovine serum albumin (BSA) for 30 min and with different concentrations of biotin-labeled peptides (0, 2, 4, 8, 16, 32, 64, 128, and 256 μM) at 4 °C for 1 h. After washing, the cells were incubated with horseradish peroxidase-conjugated neutravidin (1:10,000 dilution; ThermoFisher Scientific, Waltham, MA) for 30 min at room temperature. Enzymatic activity was detected using a 3,3′,5,5′-tetramethylbenzidine substrate (ThermoFisher Scientific), and the reaction was stopped using 2 M sulfuric acid. The absorbance at 450 nm was measured in each well using a microplate reader (Tecan, Mannedorf, Switzerland). The K_D values were then calculated using GraphPad Prism 6 software (GraphPad Inc., La Jolla, CA).

Enzyme-linked immunosorbent assay (ELISA) of peptide binding to recombinant proteins

ELISA plates were coated overnight with 100 μL of recombinant human CD44v6-Fc and CD44-Fc proteins (R&D Systems, Minneapolis, MN) at a concentration of 1 μg/mL in Tris-buffered saline with 0.05% Tween-20. BSA was used as a control. After coating, plates were incubated with 10 μM biotin-labeled CD44v6-binding peptides at 4 °C for 1 h. After washing, the cells were incubated with horseradish peroxidase-conjugated neutravidin (1:10,000 dilution; ThermoFisher Scientific) for 30 min at room temperature. Detection of enzymatic activity and measurement of absorbance was performed as described above. Relative binding was calculated based on the absorbance, in which the absorbance of BSA was set to 1.

Supplementary Table S1. Amino acid sequences of peptides selected by phage-displayed peptide library screening against CD44v6-expressing cells.

Phage clone No.	Amino acid sequence	Frequency
4R3	CNLNTIDTC	1/70
4R2	CNEWQLKSC	1/70
3R3	CAQRPMKRC	1/70
4R13	CMDVNTFSC	1/70
3R18	CHELSHESC	1/70
4R19	CNDTTFTMC	1/70
4R1	CSKLTKSKC	1/70
4R25	CYNAYMNTC	1/70
4R34	CRGVKTNRC	1/70
4R9	CLRNVTQKC	1/70
4R10	CKLMMPADC	1/70
4R11	CVNMGGIPC	1/70
4R22	CEEHCINVC	1/70
4R33	CSDMMPIDC	1/70
5R17	CIQKSITKC	1/70
5R36	CIAPRKSKC	1/70
5R12	CKLSKKTTC	1/70

Seventy clones were randomly picked after the third, fourth, and fifth rounds of screening and subjected to DNA sequencing. Seventeen phage clones with the CX7C amino acid sequences are listed. The frequency represents the occurrence of each sequence among the sequences displayed by the seventy clones.

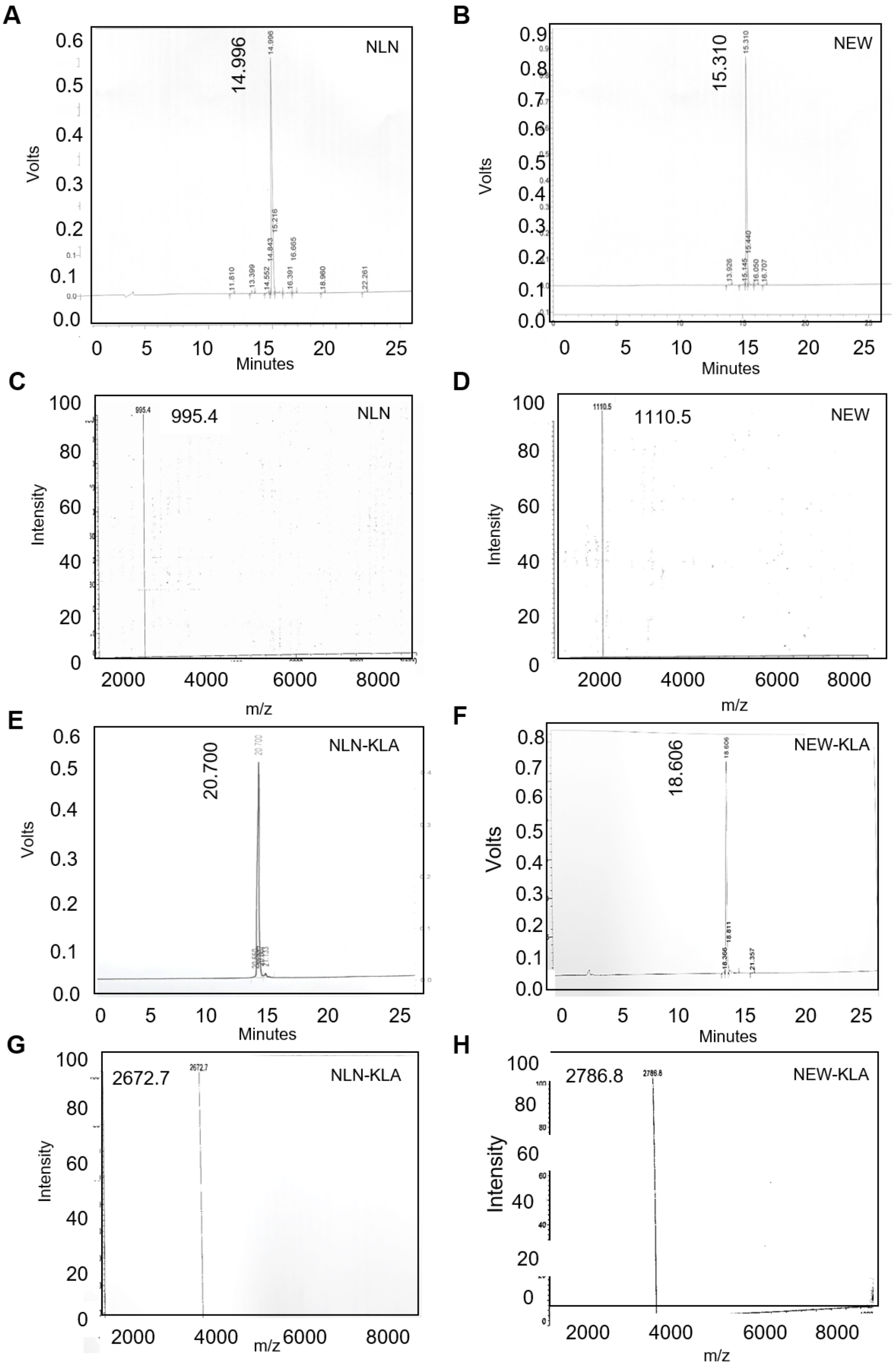
A
NLN
14.996
Volts
Minutes
B
NEW
15.310
Volts
Minutes
C
NLN
995.4
Intensity
m/z
D
NEW
1110.5
Intensity
m/z
E
NLN-KLA
20.700
Volts
Minutes
F
NEW-KLA
18.606
Volts
Minutes
G
NLN-KLA
2672.7
Intensity
m/z
H
NEW-KLA
2786.8
Intensity
m/z

Supplementary Figure S1. High-performance liquid chromatography (HPLC) chromatograms and matrix-assisted laser desorption/ionization-time of flight (MALDI-TOF) spectra of synthetic peptides. (A–B) HPLC chromatograms of NLN (A) and NEW (B). (C–D) MALDI-TOF spectra of NLN (M.W. 995.4) (C) and NEW (M.W. 1110.5) (D). (E–F) HPLC chromatograms of NLN-KLA (E) and NEW-KLA (F). (G–H) MALDI-TOF spectra of NLN-KLA (M.W. 2672.7) (G) and NEW-KLA (M.W. 2786.8) (H).

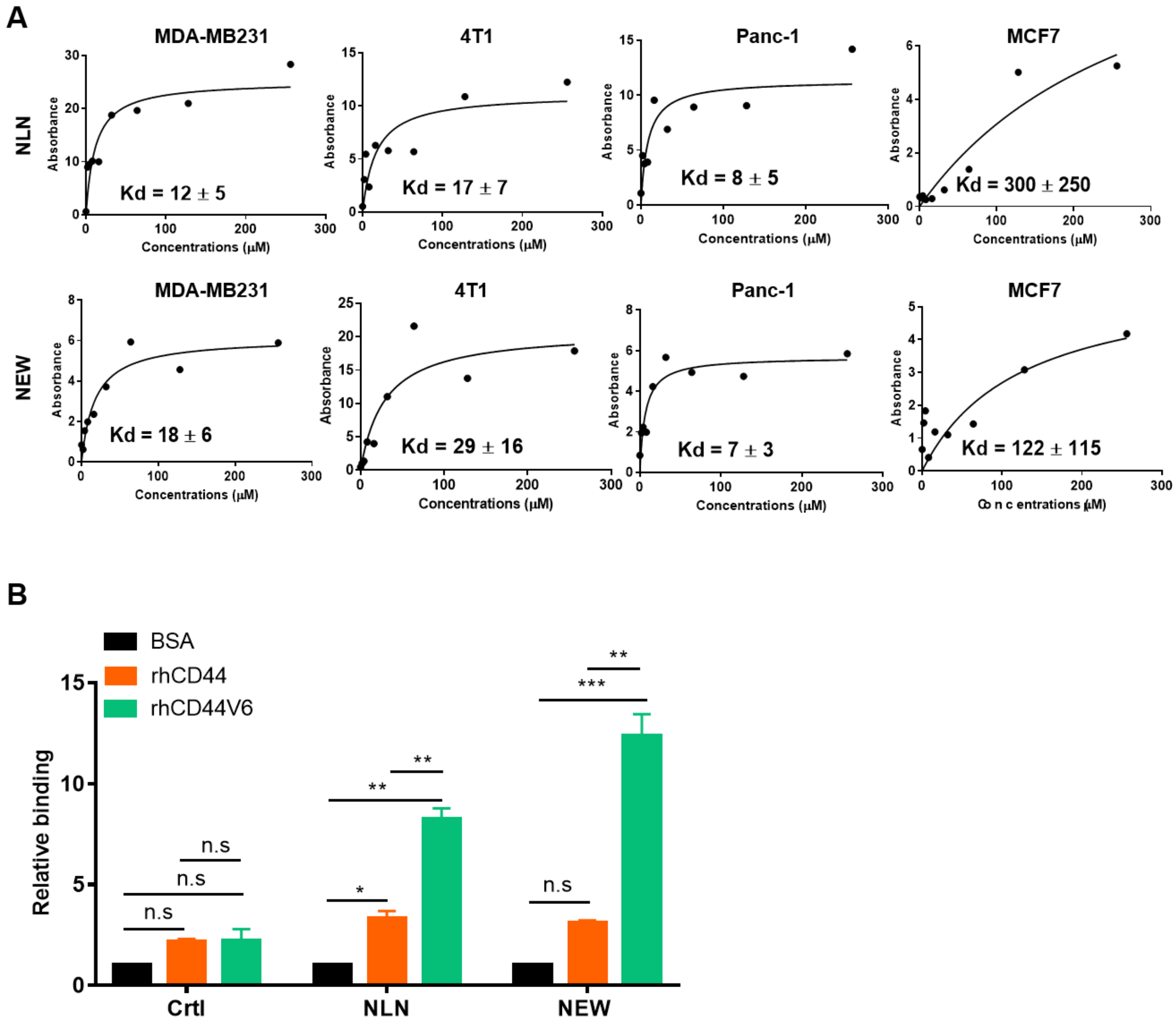

Supplementary Figure S2. Cell binding affinity and relative binding to CD44v6 protein of NLN and NEW. (A) Saturation cell binding assays to determine the binding affinities (K_D values) for the binding of NLN and NEW to MDA-MB231, 4T1, Panc-1, and MCF7 cells. (B) Relative binding of NLN and NEW to recombinant human CD44v6-Fc and CD44-Fc proteins coated on plates. BSA was used as control and set to 1. **, $P < 0.01$; ***, $P < 0.001$; n.s, not significant compared with the control peptide (Ctrl) by one-way ANOVA.

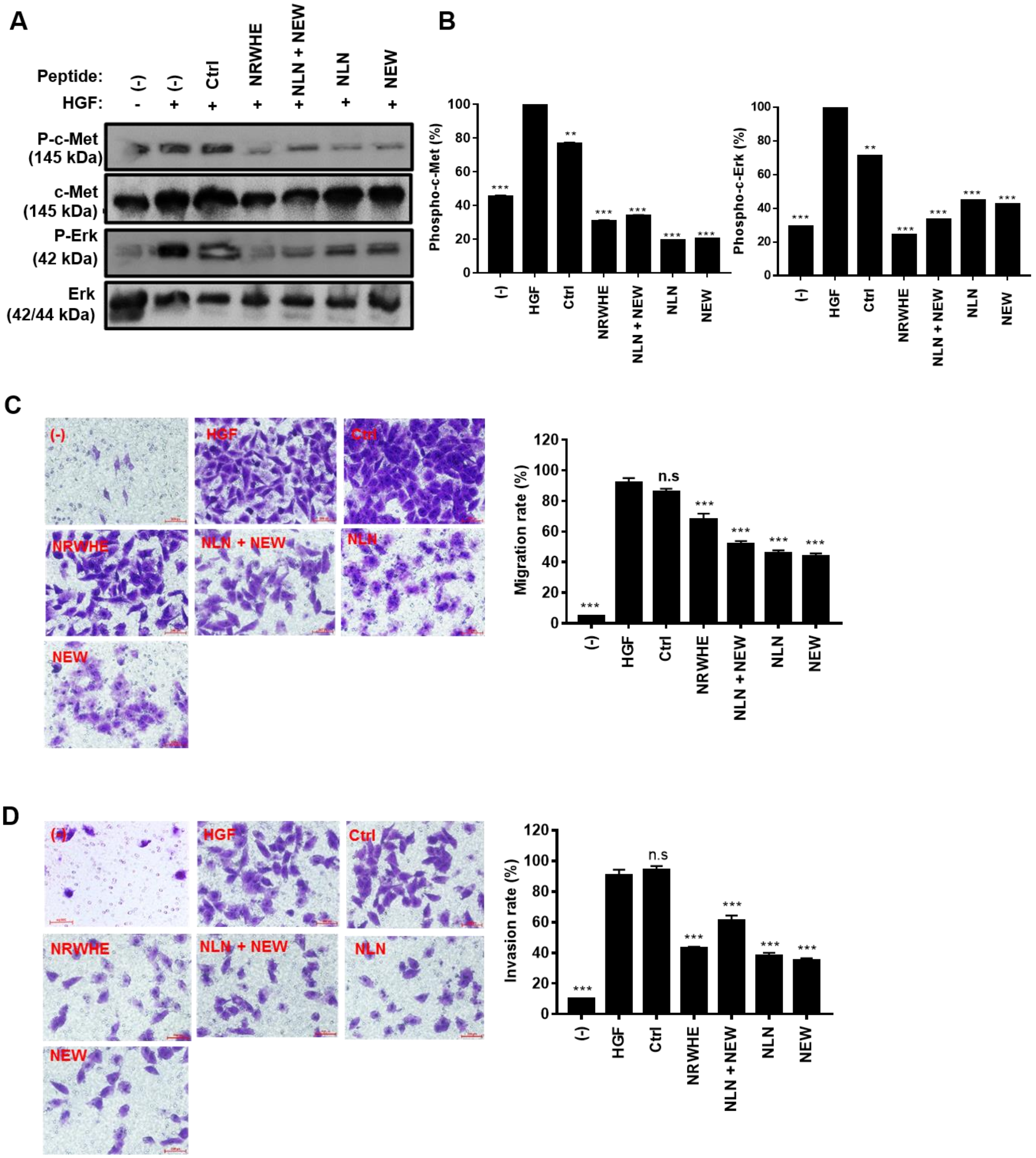

Supplementary Figure S3. NLN and NEW inhibit HGF-induced c-Met phosphorylation and cell migration and invasion in 4T1 breast tumor cells. (A) Western blotting analysis of c-Met and Erk phosphorylation in 4T1 cells pretreated or not with 5 μg/mL NLN and NEW for 10 min and treated with 25 ng/mL HGF for 10 min. (B) The quantification of c-Met and Erk

phosphorylation in 4T1 cells. (C-D) Transwell migration (C) and invasion (D) assays of 4T1 cells pre-treated or not with 5 μg/mL NLN and NEW for 10 min and subsequently treated with 25 ng/mL HGF for 10 min, followed by incubation for 24 h. Scale bars = 20 μm. Graphs (right panels) represent the quantification of the cell numbers in ten randomly selected fields. Data are shown as the means ± S.E. of three independent experiment. **, $P < 0.01$; ***, $P < 0.001$; n.s, not significant compared with HGF by one-way ANOVA.

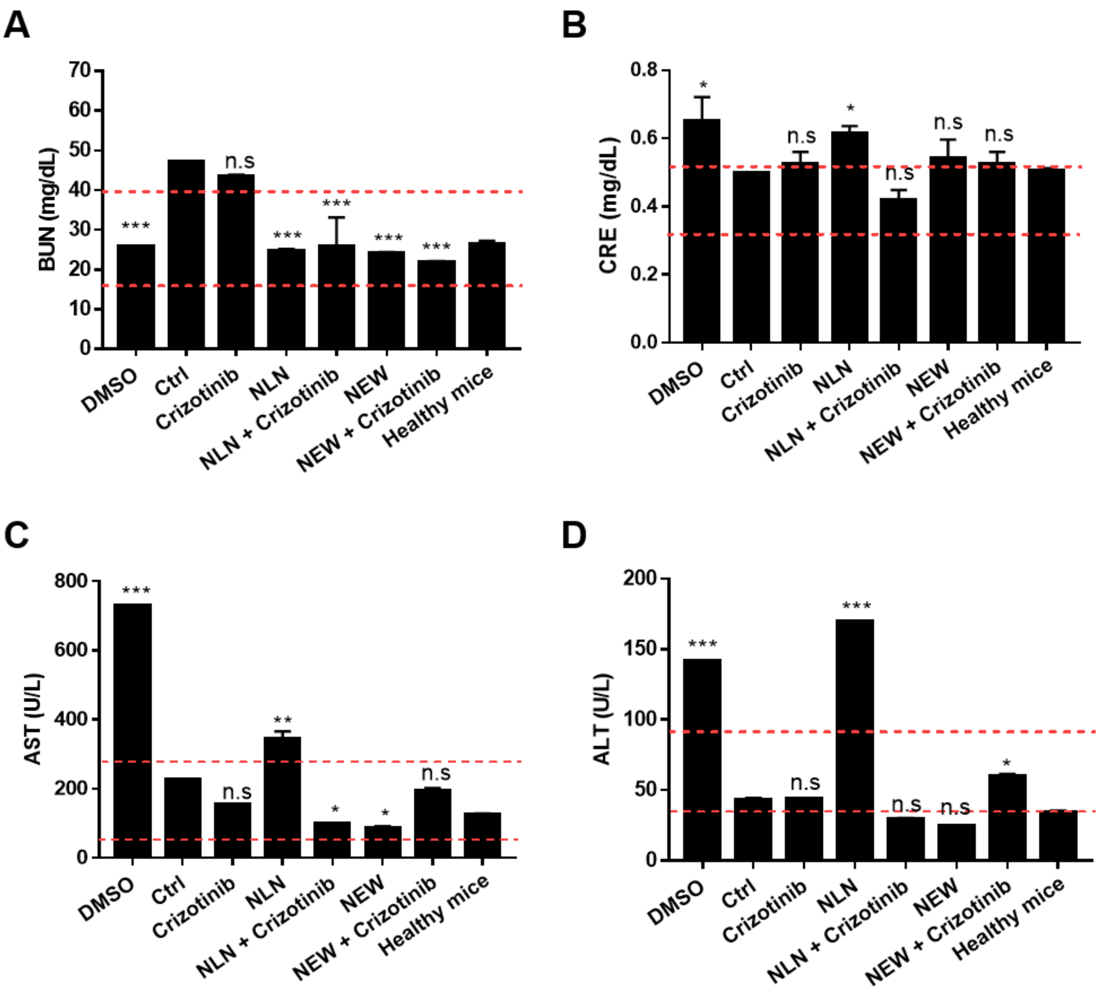

Supplementary Figure S4. Analysis of hematological parameters in MDA-MB231 experimental breast tumor metastasis model after treatment with NLN and NEW. Blood was collected from tumor-bearing BALB/c nude mice after the indicated treatments, and serum samples were prepared for analysis. (A) Blood urea nitrogen (BUN) levels. (B) Serum creatinine (CRE) levels. (C) Serum aspartate transferase (AST) levels. (D) Serum alanine transferase (ALT) levels. Dotted lines represent the normal range of each parameter of BALB/c nude mouse. Data are shown as the means ± S.E. (*n* = 5/group). *, $P < 0.05$; **, $P < 0.01$; ***, $P < 0.001$; n.s, not significant compared with the control peptide (Ctrl) by one-way ANOVA. DMSO, dimethyl sulfoxide.

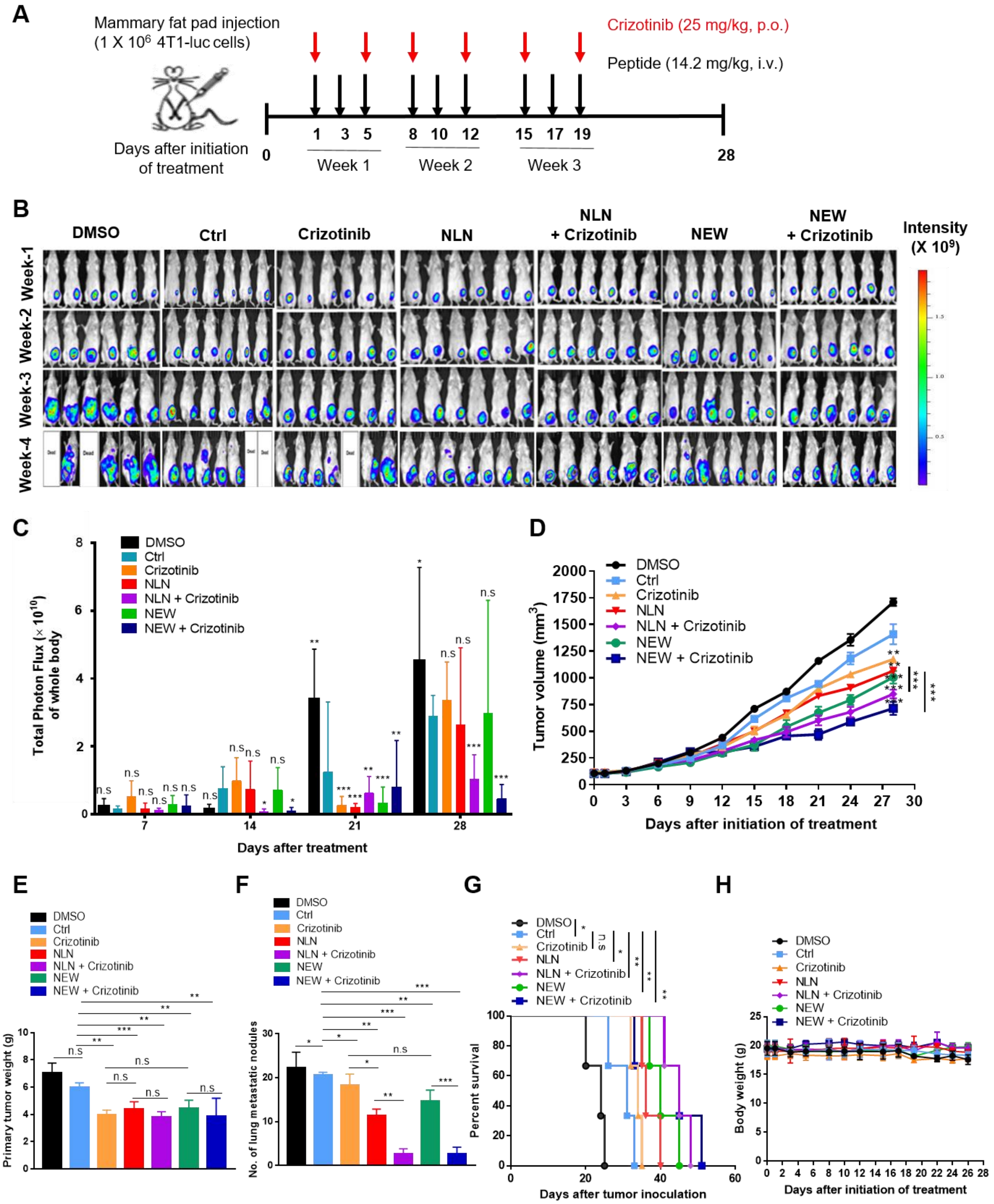

Supplementary Figure S5. NLN and NEW inhibit the primary tumor growth and metastasis in 4T1 spontaneous breast tumor metastasis model. (A) Treatment protocols. 4T1 cells were implanted into mouse mammary fat pads. Two weeks later, the BALB/c mice received

intravenous injections of NLN and NEW and orally administered crizotinib at the indicated time points (arrows, thrice weekly for 3 weeks). Mice were maintained after the treatment period to monitor the survival rates. (B) Bioluminescence imaging and tumor progression monitoring in mice treated with either NLN or NEW and crizotinib alone or in combination. (C) Quantification of the total photon flux (number of photons/second) in the whole body. (D) Tumor volumes. (E) Primary tumor weights. (F) Numbers of metastatic tumor nodules in the lungs. (G) Survival rates. (H) Body weights. Data are shown as the means ± S.E. (n = 6/group). *, $P < 0.05$; **, $P < 0.01$; ***, $P < 0.001$; n.s, not significant compared with the control peptide (Ctrl) by one-way ANOVA.

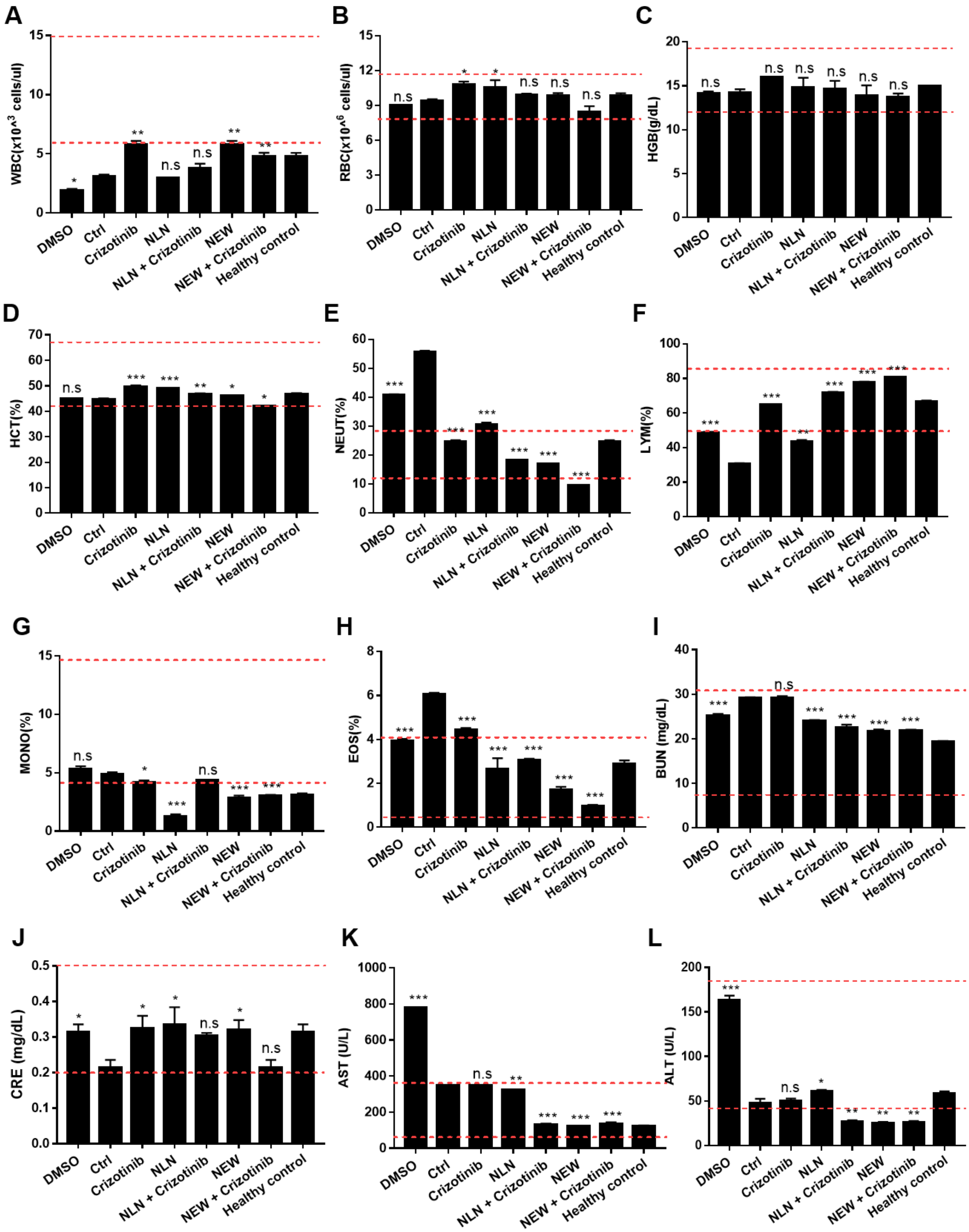

Supplementary Figure S6. Analysis of hematological parameters after treatment with NLN

and NEW in 4T1 spontaneous breast tumor metastasis model. Blood was collected from BALB/c mice bearing tumor after the indicated treatments, and serum and plasma samples were prepared for analysis. (A) White blood cell (WBC) counts. (B) Red blood cell (RBC) counts. (C) Hemoglobin (HGB) levels. (D) Hematocrit (HCT) levels. (E) Neutrophil (NEUT) counts. (F) Lymphocyte (LYM) counts. (G) Monocyte (MONO) counts. (H) Eosinophil (EOS) counts. (I) Blood urea nitrogen (BUN) levels. (J) Serum creatinine (CRE) levels. (K) Serum aspartate transferase (AST) levels. (L) Serum alanine transferase (ALT) levels. Dotted lines represent the normal range of each parameter of BALB/c mouse. Data are shown as the means ± S.E. (n = 3/group). *, $P < 0.05$; **, $P < 0.01$; ***, $P < 0.001$; n.s, not significant when compared with the control peptide (Ctrl) by one-way ANOVA. DMSO, dimethyl sulfoxide.

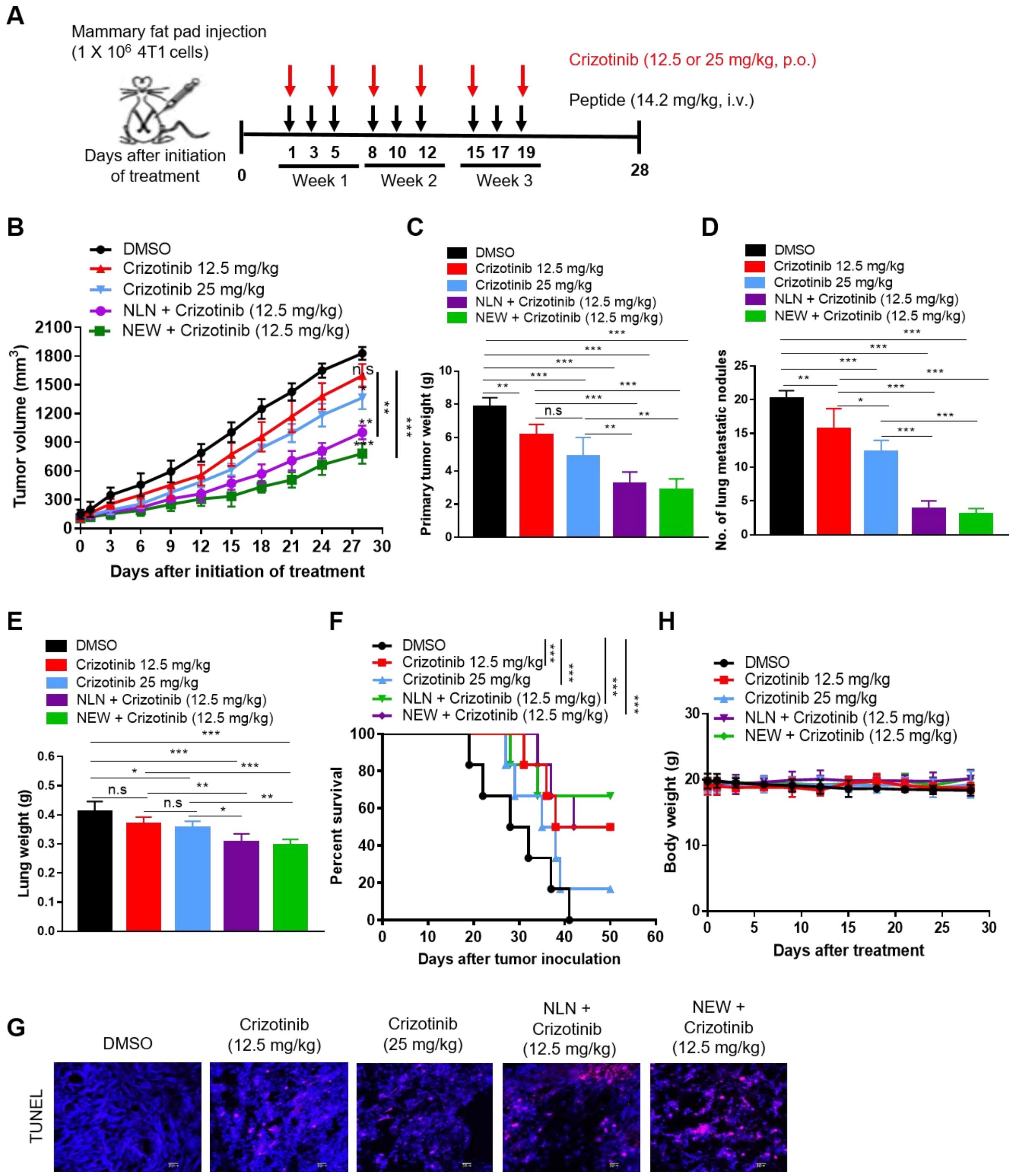

Supplementary Figure S7. Combined treatment of either NLN or NEW and crizotinib reduces the therapeutic dose of crizotinib in 4T1 spontaneous breast tumor metastasis model. (A) Treatment protocols. 4T1 cells were inoculated into mouse mammary fat pads. Two weeks

later, the BALB/c mice received intravenous injections of NLN and NEW (14.2 mg/kg body weight, black arrows, thrice weekly for three weeks) and orally administered crizotinib (12.5 and 25 mg/kg body weight, red arrows, twice weekly for three weeks). (B) Tumor volumes. (C) Primary tumor weights. (D) Numbers of metastatic tumor nodules in the lungs. (E) Lung weights. (F) Survival rates. (G) TUNEL staining (red) in primary tumor tissues. Nuclei were stained with DAPI (blue). Scale bars = 20 μm. (H) Body weights. Data are shown as the means ± S.E. (n = 10/group). *, $P < 0.05$; **, $P < 0.01$; ***, $P < 0.001$; n.s, not significant compared with the control peptide (Ctrl) by one-way ANOVA.

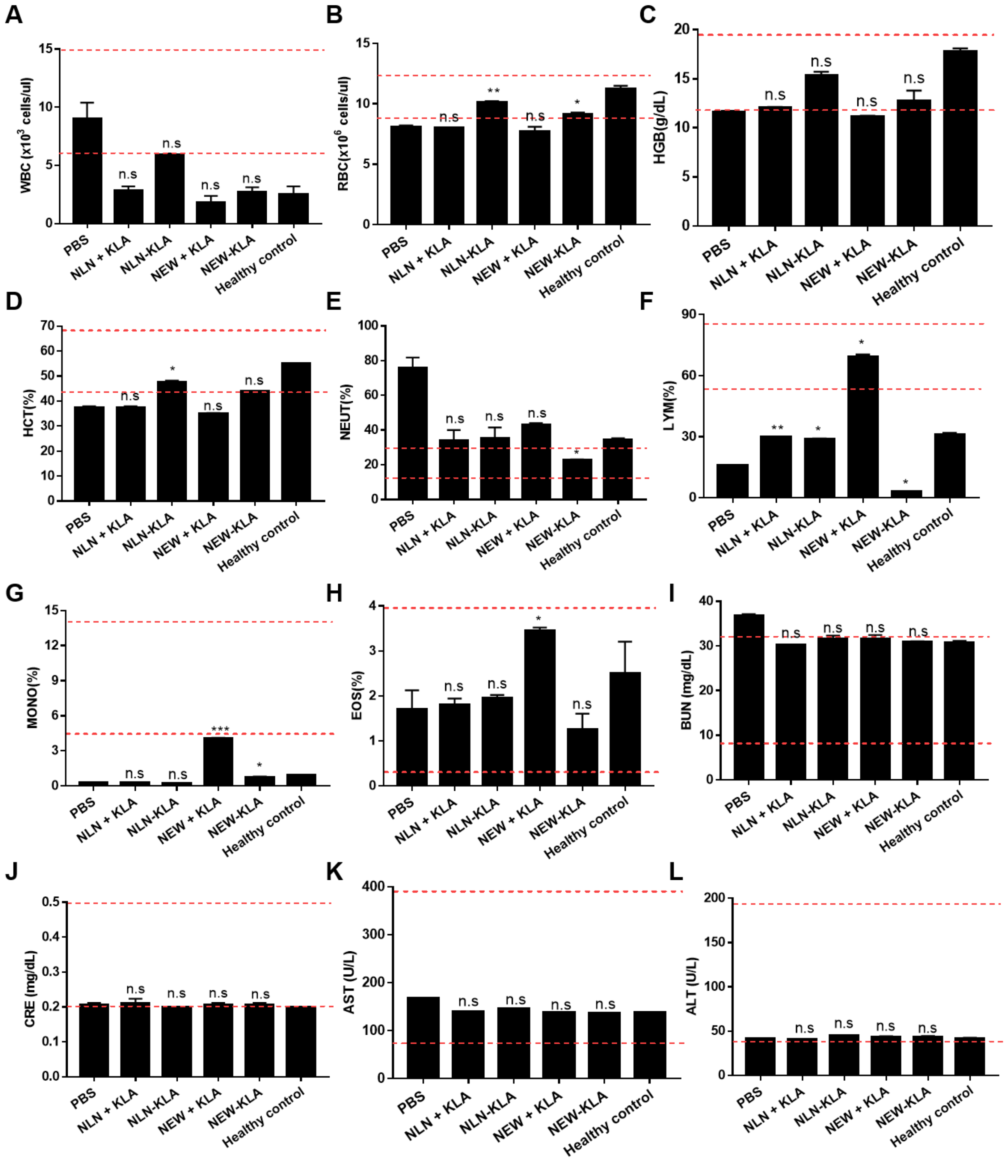

Supplementary Figure S8. Analysis of hematological parameters after treatment with NLN-KLA and NEW-KLA in 4T1 spontaneous breast tumor metastasis model. Blood was collected from tumor-bearing BALB/c mice after the indicated treatments, and serum or plasma

samples were prepared for analysis. (A) White blood cell (WBC) counts. (B) Red blood cell (RBC) counts. (C) Hemoglobin (HGB) levels. (D) Hematocrit (HCT) levels. (E) Neutrophil (NEUT) counts. (F) Lymphocyte (LYM) counts. (G) Monocyte (MONO) counts. (H) Eosinophil (EOS) counts. (I) Blood urea nitrogen (BUN) levels. (J) Serum creatinine (CRE) levels. (K) Serum aspartate transferase (AST) levels. (L) Serum alanine transferase (ALT) levels. Dotted lines represent the normal range of each parameter of BALB/c mouse. Data are shown as the means ± S.E. (n = 5/group).*, $P < 0.05$; **, $P < 0.01$; ***, $P < 0.001$; n.s, not significant compared with the phosphate-buffered saline (PBS) by one-way ANOVA.

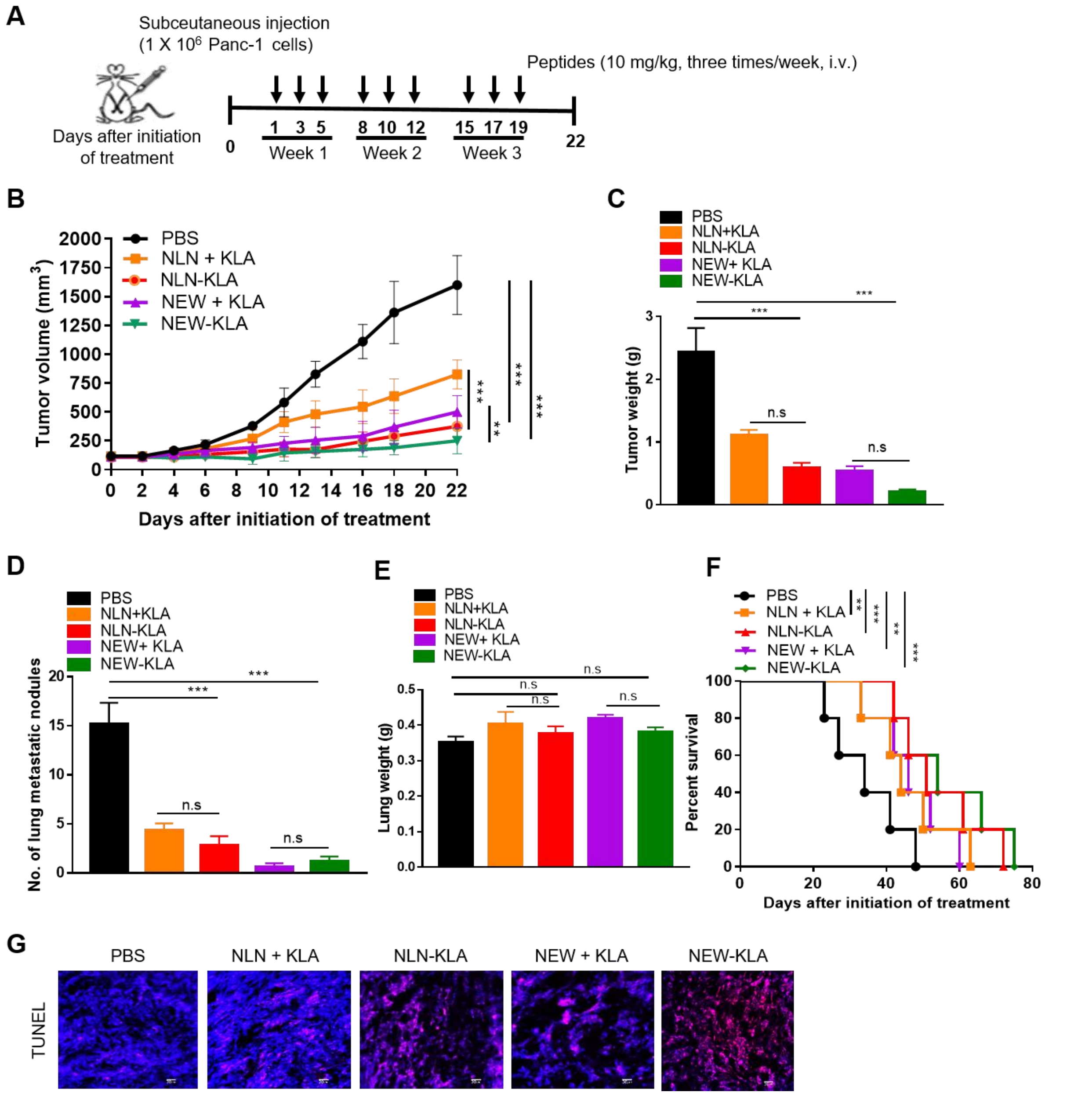

Supplementary Figure S9. NLN-KLA and NEW-KLA inhibit the tumor growth and metastasis in Panc-1 spontaneous pancreatic tumor metastasis model. (A) Panc-1 cells were injected subcutaneous into BALB/c nude mice. Two weeks later, mice were intravenously injected with NLN-KLA, NEW-KLA, combination of NLN and KLA (NLN + KLA), or combination of NEW and KLA (NEW + KLA) at the indicated time points (arrows, thrice weekly for 3 weeks).

(B) Tumor weights (C) Number of metastatic tumor nodules in the lungs. (D) Lung weights (E) Percent survival. (F) TUNEL staining (red) in primary tumor tissues. Nuclei were stained with DAPI (blue). Scale bars = 20 μm. Data are shown as the means ± S.E. ($n = 10$/group). **, $P < 0.01$; ***, $P < 0.001$; n.s, not significant compared with the phosphate-buffered saline (PBS) by one-way ANOVA.
